# Age-related late-onset disease heritability patterns and implications for genome-wide association studies

**DOI:** 10.7717/peerj.7168

**Published:** 2019-06-14

**Authors:** Roman Teo Oliynyk

**Affiliations:** 1 Centre for Computational Evolution, University of Auckland, Auckland, New Zealand; 2 Department of Computer Science, University of Auckland, Auckland, New Zealand

**Keywords:** GWAS, Genetics, Polygenic risk, Heritability, Genomics, Model, Late-onset disease, Age, Simulation, SNP, Variant

## Abstract

Genome-wide association studies (GWASs) and other computational biology techniques are gradually discovering the causal gene variants that contribute to late-onset human diseases. After more than a decade of genome-wide association study efforts, these can account for only a fraction of the heritability implied by familial studies, the so-called “missing heritability” problem. Computer simulations of polygenic late-onset diseases (LODs) in an aging population have quantified the risk allele frequency decrease at older ages caused by individuals with higher polygenic risk scores (PRSs) becoming ill proportionately earlier. This effect is most prominent for diseases characterized by high cumulative incidence and high heritability, examples of which include Alzheimer’s disease, coronary artery disease, cerebral stroke, and type 2 diabetes. The incidence rate for LODs grows exponentially for decades after early onset ages, guaranteeing that the cohorts used for GWASs overrepresent older individuals with lower PRSs, whose disease cases are disproportionately due to environmental causes such as old age itself. This mechanism explains the decline in clinical predictive power with age and the lower discovery power of familial studies of heritability and GWASs. It also explains the relatively constant-with-age heritability found for LODs of lower prevalence, exemplified by cancers.

## Introduction

Throughout the ages, late-onset diseases (LODs) were considered the bane of the lucky few who survived to an advanced age. Over the last couple of centuries, however, continuous improvements in sanitation, life and work environments, vaccinations, disease prevention, and medical interventions have extended the average life expectancy by decades.

With a growing fraction of the population being of advanced age, the leading causes of mortality are now heart disease, cancer, respiratory disease, stroke, and notably Alzheimer’s disease (AD) and other dementias (Murphy et al., 2017). The need—and with it, the effort being made—to determine the causes of LODs is ever increasing, and one of the targets of medicine has become combating aging itself in addition to specific age-related diseases (Franceschi et al., 2018).

One of the major goals of computational biology is to identify gene variants that lead to increased odds of LODs. Nevertheless, polygenic LODs remain resistant to the discovery of sufficient causal gene variants that would allow for accurate predictions of an individual’s disease risk (Manolio et al., 2009; Clarke & Cooper, 2010; Kumar et al., 2016). This is despite the fact that LODs with varied symptoms and phenotypes show high heritability in twin and familial studies (Zaitlen & Kraft, 2012).

At a young age, the human organism usually functions as well as it ever will. With time, the organism’s functions decline, leading to the common image of aging as one of thinning hair and a loss of pigmentation in what remains, increased wrinkling and altered pigmentation of the skin, reductions in height, muscle and bone mass, joint pain, and deficits in hearing, sight, and memory (Fedarko, 2018). The combination of genetic liability, environmental factors, and the physiological decline of multiple organism systems leads to individual disease presentation. Genetic variation may be either protective or detrimental when compared to the average distribution of common gene variants that defines human conditions as it applies to polygenic LODs.

Researchers engaged in genome-wide association studies (GWASs) often set an unrealistic expectation that a combination of causal single nucleotide polymorphisms (SNPs)—also known as a polygenic score—will, irrespective of the patient’s age, completely predict an individual’s predisposition to an LOD to a degree matching the maximum heritability found in familial studies (Naj, Schellenberg & Alzheimer’s Disease Genetics Consortium (ADGC), 2017; Silva et al., 2015). The lost heritability debate, in the case of LODs, often treats polygenic LODs as if they were binary hereditary phenotypic features rather than facets of failure processes that arise in the human body (Oh, Lee & Wagers, 2014) when it is past its reproductive prime and when evolutionary selection is significantly relaxed compared to younger ages (Fedarko, 2018).

Genome-wide association studies can implicate a subset of SNPs that can typically explain between 10% and 20% of the genetic heritability of a polygenic LOD (Visscher et al., 2017). There are two complementary hypotheses explaining this so-called missing heritability (Eyre-Walker, 2010; Yang et al., 2012; Thornton, Foran & Long, 2013; Agarwala et al., 2013). The first is the hypothesis that LODs are caused by a combination of a large number of relatively common alleles of small effect (Goldstein, 2009). GWASs have been able to discover only a small number of moderate-effect SNPs, but a large number of SNPs remain below GWASs’ statistical discovery power. The second hypothesis states that LODs are caused by a relatively small number of rare, moderate- or high-effect alleles with a frequency below 1% that likely segregate in various proportions into subpopulations or families (Dickson et al., 2010; North & Beaumont, 2015) and are also under the radar of GWASs’ discovery power.

Both scenarios can contribute to observational facts, but their relative weights vary depending on the genetic architecture of an LOD (Park et al., 2011). Rare highly detrimental alleles become indistinguishable in their presentation from the OMIM cataloged conditions and will likely be diagnosed as a separate disease or syndrome. The population age distribution and individual disease progression of polygenic LODs are best understood by considering the aging process itself as an ongoing loss of function, which can be modulated by the genetic liabilities resulting from both common and rare SNP distributions combined with changing environmental and lifestyle variables. It has been determined (Anderson et al., 2011; Yang et al., 2015) that common variants very likely explain the majority of heritability for most complex traits.

While the findings of GWASs can explain only a fraction of heritability, the systematically collected SNP correlations provide a good indication of what to expect regarding the effect sizes and allele frequency distribution of as yet undiscovered SNPs (Eyre-Walker, 2010). Many studies focus on constructing hypotheses, defining the types of gene variants that could explain the missing heritability, proving why these gene variants are difficult to discover, and identifying the evolutionary processes that led to the hypothesized and observed gene variant distributions (Manolio et al., 2009; Clarke & Cooper, 2010; So et al., 2011; Zaitlen & Kraft, 2012; Thornton, Foran & Long, 2013; Wood et al., 2014). These studies explore the effect sizes and allele frequencies that GWAS would expect to find for LODs as well as the genetic architecture of complex traits and their implications for fitness.

The age-related heritability decline of some LODs has been assumed for decades. The precise magnitude of heritability change with age is typically unknown for most LODs, and the effects are not understood and often ignored or overlooked. Most GWASs recommend homogeneity in cohort age—that is, that the same age window should be targeted—although it has been suggested (Li & Meyre, 2013) that individuals with an early age of onset are likely to have greater genetic susceptibility. Discussing a replication study design, Li & Meyre (2013) stated, “Once the risk of false positive association has been ruled out by initial replication studies, the focus of the association can be extended to different age windows.” Another common approach is to “age adjust” the effect (Zaitlen et al., 2012) with the goal of removing or averaging out the effect of aging rather than examining its consequences more thoroughly. Two recent studies (Lin et al., 2014; Bjørnland et al., 2018) emphasize the need to explore “extreme phenotype sampling” in order to improve GWAS discovery, including using cohorts that are diverse in age.

One of the first geneticists to build a conceptual foundation for disease susceptibility, and the pioneer of the liability threshold approach, was D. S. Falconer in his studies of inheritance estimated from the prevalence among relatives (Falconer, 1965) and his 1967 follow-up study exploring the prevalence patterns of LODs, specifically diabetes (Falconer, 1967), and their decreasing heritability with age. These concepts were not followed up by systematic research, likely due to the difficulties involved in setting up large familial studies and perhaps the perceived limited clinical use of this kind of expensive and time-consuming project.

Detailed, high-granularity data on heritability by age are rare for most diseases. The familial heritability, clinical, and epidemiological statistics were available for eight prevalent LODs, AD, type 2 diabetes (T2D), coronary artery disease (CAD), and cerebral stroke, and four late-onset cancers: breast, prostate, colorectal, and lung cancer. These statistics served as the basis for this study’s analysis and conclusions. This study investigated the model in which the polygenic risk of an individual remains constant with age and endeavored to establish how the higher odds of becoming ill of individuals with higher polygenic liability may lead to a change of risk allele distribution as the population ages and whether this alone may explain some of the known observational facts.

A set of computer simulations quantified the change in the risk allele representation for these LODs as the population ages and determined how and why these changes affect clinical predictive power and GWAS statistical discovery power with age more for some LODs than for others.

## Methods

### The model definition

According to Chatterjee, Shi & Garca-Closas (2016), the conditional age-specific incidence rate of the disease, *I*(*t*|*G*) that is defined as the probability of developing the disease at a particular age *t*, given that a subject has been disease-free until that age, can be modeled using Cox’s proportional hazards model (Cox, 1972):

(1) $$I(t|G) = {I_0}(t) \cdot {\rm{exp}}\left(\sum\limits_k {b_k}{G_k}\right),$$

where $G = ({G_1}, \ldots, {G_k})$ is the multiplicative effect of a set of risk factors on the baseline hazard of the disease *I*_0_(*t*). The set of age-independent variables in *G* could include genetic and environmental risk factors, as well as their interaction terms.

The following summary from Chatterjee, Shi & Garca-Closas (2016) is particularly relevant to the methodology of this research: “logistic regression methods are preferred for the evaluation of multiplicative interactions. For case-control studies, if it can be assumed that environmental risk factors are independent of the SNPs in the underlying population, then case-only and related methods can be used to increase the power of tests for gene-environment interactions. To date, post-GWAS epidemiological studies of gene-environment interactions have generally reported multiplicative joint associations between low-penetrant SNPs and environmental risk factors, with only a few exceptions.” This means that the polygenic score $G = \sum\nolimits_k {b_k}{G_k}$, as the lifelong characteristic of each individual, is used multiplicatively with *I*_0_(*t*), which encompasses environmental and aging effects.

It is important to note that the simulations conducted in this research rely on the model genetic architectures of the analyzed LODs, not a complete GWAS map of their experimentally discovered SNPs, because GWAS-discovered sets can explain only a fraction of these LODs’ heritability. These model genetic architecture SNPs are treated as “true” causal variants for disease liability and heritability, as discussed in Chatterjee, Shi & Garca-Closas (2016), rather than GWAS-linked SNPs. They are used as a priori known constant causal SNPs that combine into individual polygenic risk scores (PRSs) for an LOD, as will be described further. The study by Pawitan, Seng & Magnusson (2009), which followed the mathematical foundation and simulational validation of the liability model developed in Noh et al. (2006), served as a basis for the genetic architectures used in this study.

Taking an aging population simulation approach allows for the identification of individuals becoming ill and, with them, the corresponding allele distribution between cases and controls, without intermediate steps and operating directly with the odds-ratio-based PRSs common to GWASs and clinical studies. The core of the simulation is Algorithm 1, operating on the known yearly incidence of an LOD and the PRSs for all individuals based on a modeled LOD genetic architecture:

**Algorithm 1:** Sampling individuals diagnosed with a disease proportionately to their polygenic odds ratio and incidence rate.

**for** *age* = 1 *to MaxAge* **do**
*numberIllThisYear* = *I*(*age*)·*N* // *N* is unaffected population
**for** *i* = 1 *to numberIllThisYear* **do**
*HRsum* = 0 // will recalculate sum of all HRs
**for** *u* = 1 *to N* **do**
*HRsum* = *HRsum*+*ORtoHR*(*G_u_*) // calculate the HR total
*LOOKUP*(*add*,*HRsum*,*u*) // add *u_th_* individual to the lookup table
**end**
*rand* = *RandomNumber*(0,*HRsum*) // pick a random number
*ill* = *LOOKUP*(*find*, *rand*,*N*) // found newly diagnosed
*N* = *N* −1 // decrement in number of healthy individuals
*ProcessAndAnalyze*(*ill*)
**end**
**end**
Note: an individual makes a sampling target proportionate to the hazard ratio (HR) in the *LOOKUP*() table. Odds ratios (ORs) are converted to HRs, similar to the approach taken by Wang (2013). An individual with an HR of 15 will be 150 times more likely to be sampled than an individual with an HR of 0.1. *ProcessAndAnalyze*() moves newly diagnosed individual from the healthy to the ill population pool, accounts for allele distribution, case/control ORs, etc.

Descriptively, the algorithm works as follows. In this prospective simulation, each next individual to be diagnosed with an LOD is chosen proportionately to that individual’s relative PRS at birth relative to all other individuals in the as-yet-unaffected population. The number of individuals diagnosed annually is determined using the model incidence rate curve derived from clinical statistics. In this manner, the aging process is probabilistically reproduced using a population simulation model rather than a computational model. As the simulation progresses, the risk alleles are tracked for all newly diagnosed individuals and the remaining unaffected population, and their representation in the affected and remaining population is statistically analyzed.

The following sections describe the model genetic architectures, the LOD incidence models and the statistical foundations of this research.

### Allele distribution models

An in-depth review by Pawitan, Seng & Magnusson (2009) extensively analyzed models of genetic architecture and through simulations determined the number of alleles required to achieve specific heritability and estimated the discovery power of GWASs. They calculated allele distributions and heritability and ran simulations for six combinations of effect sizes and minor allele frequencies (MAFs). Reliance on the conclusions of Pawitan, Seng & Magnusson (2009) in this research makes it unnecessary to repeat the preliminary steps of evaluating the allele distributions needed to achieve the requisite heritability levels. The Pawitan, Seng & Magnusson (2009) alleles represent the entire spectrum ranging from common, low-frequency, low-effect-size alleles to extremely rare, high-effect, high-frequency alleles. The five most relevant architectures were implemented in this study; see Table 1.

**Table 1:** Genetic architecture scenarios.

Scenario	MAF	Odds ratio
(A) Common low	0.073–0.499	1.05–1.15
(B) Modest low	0.0365–0.2495	1.05–1.15
(C) Rare low	0.0146–0.0998	1.05–1.15
(D) Rare medium	0.0146–0.0998	1.28–2.01
(E) Rare high	0.0073–0.0499	1.63–4.05

**Note:**

Allele distributions as modeled by Pawitan, Seng & Magnusson (2009).

It is also handy for repeatable allele tracking, rather than generating the continuous random spectrum of allele frequencies and effect sizes, to follow the Pawitan, Seng & Magnusson (2009) configuration and discretize the MAFs into five equally spaced values within the defined range, with an equal proportion of each MAF and an equal proportion of ORs. For example, for scenario A, the MAFs are distributed in equal proportion at 0.073, 0.180, 0.286, 0.393, and 0.500, while the OR values are 1.15, 1.125, 1.100, 1.075, and 1.05, resulting in 25 possible combinations. Having multiple well-defined alleles with the same parameters facilitated the tracking of their behaviors with age, LOD, and simulation incidence progression.

An individual PRSs β can be calculated as the sum of the effect sizes of all alleles, which is by definition a log(OR) (natural logarithm of OR) for each allele, also following Pawitan, Seng & Magnusson (2009):

(2) $${\rm{\beta }} = {\rm{log}}({\rm{OR}}) = \sum\limits_k {a_k}{\rm{log}}({\rm{O}}{{\rm{R}}_k}),$$

where *a_k_* is the number of risk alleles (0, 1, or 2) and OR_*k*_ is the ORs of additional liability presented by the *k*-th allele.

Variance of the allele distribution is determined by:

(3) $${\rm{var}} = 2\sum\limits_k {p_k}(1-{p_k})({\rm{log}}({\rm{O}}{{\rm{R}}_k}{))^2},$$

where *p_k_* is the frequency of the *k*-th genotype (Pawitan, Seng & Magnusson, 2009).

The contribution of genetic variance to the risk of the disease is heritability:

(4) $${h^2} = {{{\rm{var}}} \over {{\rm{var}} + {{\rm{\pi }}^2}/3}},$$

where π^2^/3 is the variance of the standard logistic distribution (Noh et al., 2006). For example, the number of variants needed for the Scenario A LODs is summarized in Table 2.

**Table 2:** Heritability of analyzed LODs and an example of required variant numbers for common low-effect variants: Scenario A.

	Highly prevalent LODs				Cancers			
	AD	T2D	CAD	Stroke	Prostate	Colorectal	Breast	Lung
Heritability	0.795	0.69	0.55	0.41	0.57	0.40	0.31	0.095
Variants	3,575	2,125	1,175	625	1,250	600	400	100

Following Pawitan, Seng & Magnusson (2009), the variants are assigned to individuals with frequencies proportionate to MAF *p_k_* for SNP *k*, producing, in accordance with the Hardy–Weinberg principle, three genotypes (AA, AB, or BB) for each SNP with frequencies $p_k^2$, 2*p_k_*(1 − *p_k_*) and (1 − *p_k_*)^2^. The mean value β_mean_ of the population distribution can be calculated using the following equation:

(5) $${{\rm{\beta }}_{{\rm{mean}}}} = 2\sum\limits_k {p_k} \cdot {\rm{log}}({\rm{O}}{{\rm{R}}_k})$$

Customarily, the individual PRSs are normalized relative to *G*_mean_, resulting in a zero mean initial population PRS, making it easy to compare higher- and lower-risk individuals. Figs. S13 and S14 in Article S1 depict the corresponding population distribution of detrimental variants and PRSs for the common, low-effect-size genetic architecture.

A part of simulation functionality is to allocate the genetic architectures and calculate the variance, using Eq. (3), of each genetic architecture instance described above. Each genetic architecture listing is represented in the File S1 executable folder; for example, the file “CommonLow.txt” lists the variants describing Scenario A (only three columns are used for this simulation: SNP—internal use identifier, EAF—effect allele frequency, and OR). In the case of Scenario A, var = 0.09098 for the single set of SNPs listed in this file. Rearranging Eq. (4) and changing the multiple allows for the discovery of the number of variant sets for each LOD, as seen in Table 2, and for the target heritability to be closely approximated. Each simulation run calculates the PRS variance within the population and records heritability and allele distributions for the case and control populations as the simulated age progresses.

### Evaluating GWAS statistical power

Genome-wide association studies statistical power is the estimate of the ability of GWASs to detect associations between DNA variants and a given trait, and depends on the experimental sample size, the distribution of effect sizes, and the frequency of these variants in the population (Visscher et al., 2017). Statistical power calculations are very useful in a case/control study design for determining the minimum number of samples that will achieve adequate statistical power; conventionally, statistical power of 80% is considered to be acceptable (Hong & Park, 2012). To achieve greater power, a disproportionately larger number of cases and controls may be required, which is frequently unrealistic for cohort studies. A number of statistical power calculators are available, for example, Sham & Purcell (2014). This study utilized the Online Sample Size Estimator (2018).

The progress made by GWASs over the last decade, particularly in relation to polygenic traits, was to a large extent due to ever-increasing cohort sizes. Cohort size is one of the principal factors limiting GWAS discovery power, making it an important benchmark for this study. Here, the cohort size is defined as the number of cases and controls needed to achieve 80% statistical discovery power when the case/control allele frequency changes with cohort age for a subset of representative alleles in the model genetic architectures. For each such allele in the simulated population, the allele frequency for cases and controls is tracked as age progresses. The difference between these MAFs gives the non-centrality parameter (NCP) λ for two genetic groups (Sham & Purcell, 2014; Luan et al., 2001):

(6) $${\rm{\lambda }} = N \cdot {p_1} \cdot {p_2} \cdot {({{\rm{\beta }}_1}-{{\rm{\beta }}_2})^2},$$

where *N* is the overall population sample size and *p*_1_ and *p*_2_ the fractions of cases and controls, and β_1_ and β_2_ are the case and control mean log(OR) for an allele of interest. The values *p*_1_ = *p*_2_ = 0.5, or an equal number of cases and controls, are used throughout this publication.

Having obtained NCP λ from Eq. (6), Luan et al. (2001) recommended using SAS or similar statistical software to calculate the statistical power, using the following SAS statement:

(7) $${\rm{StatPower}} = 1-{\rm{PROBF}}({\rm{FINV}}(0.99999995,1,N-4),1,N-4,{\rm{\lambda }}).$$

The conversion of this equation to its *R* equivalent, which was used to process the simulation output, is:

(8) $${\rm{StatPower}} = 1-pf(qf({\rm{PSign}},1,N-4),1,N-4,{\rm{\lambda }}),$$

where PSign = 0.99999995 corresponds to a 5 × 10^−8^ significance level. The outputs of this conversion were validated using the Online Sample Size Estimator (2018). This equation returns statistical power based on a case/control number and the NCP as calculated above. To find the number of cases needed for 80% GWAS discovery power, having the (β_1_ − β_2_), a rapid convergence *R* routine was used to iterate the values of *N* until the value of StatPower matched 0.8 (80%) with an accuracy better than *±*0.01% for each age and allele distribution of interest.

### LOD incidence functional approximation

Chapter S3 in Article S1 describes the functional approximations of the yearly incidence of AD, T2D, CAD, and cerebral stroke, and four late-onset cancers: breast, prostate, colorectal, and lung cancer. As a short summary, for all of the above LODs, the incidence rate curves can be approximated during the initial disease onset periods with an annual incidence growth that is close to exponential. This exponential growth continues for decades; see Table 3 and Chapter S3 in Article S1.

**Table 3:** Age to which LOD incidence rate rises exponentially.

	Highly prevalent LODs				Cancers			
	AD	T2D	CAD	Stroke	Prostate	Colorectal	Breast	Lung
Age (years)	103	55	81	79	48	62	72	70

Later, the growth may flatten in old age, as is the case with T2D, slow down, as is the case with cerebral stroke and CAD, or continue exponentially to a very advanced age, as is the case with AD. An *R* script automates the determination of the best fit for logistic and exponential approximation from the clinical incidence data.

### Sampling based on the LOD incidence rate and individual PRS

The incidence rate functional approximations for the analyzed LODs are used to find the average number of diagnosed individuals *N_d_* for each year of age *t* as a function of the incidence rate *I*(*t*) and the remaining population unaffected by the LOD *N_u_*(*t*) in question:

(9) $${N_d}(t) = I(t) \cdot {N_u}(t),$$

In the next year of age, the unaffected population will have been reduced by the number of individuals diagnosed in the previous year *N_d_*(*t*):

(10) $${N_u}(t + 1) = {N_u}(t)-{N_d}(t) = {N_u}(t)(1-I(t)).$$

The number of individuals projected to become ill per year, as well as the remaining unaffected population, is applied in Algorithm 1.

For the PRS of the simulated population based on ORs built using the Pawitan, Seng & Magnusson (2009) model, if an LOD is characterized by low incidence within an age interval, and the OR is close to 1, OR values are practically identical to HR or relative risk (RR). For example, Song et al. (2014) treat OR and RR as equivalent in the case of breast cancer in their simulation study. For higher values, an OR usually significantly exceeds the RR. An adjustment formula by Zhang & Kai (1998) can provide OR to HR approximation.

### Individual values analysis and cohort simulation

It can be expected that, for an LOD with higher incidence and heritability, the fraction of the highest-PRS individuals will diminish more rapidly with age. For such LODs, the relatively-lower-PRS individuals will represent the bulk of the LOD cases at an earlier age compared to LODs with lower incidence and heritability. The LODs are characterized by a wide range of heritability and progression patterns of incidence rate with age. For example, T2D and breast cancer begin their incidence rise relatively early but reach quite different levels at older age, while colon cancer and AD start later and also reach quite different maximum incidence and cumulative incidence levels; see Fig. S1 in Article S1. In the absence of mortality, both due to general frailty and other LODs, the incidence progression makes it appear as though, sooner or later, depending on the incidence magnitude, the majority of the population would be diagnosed with every LOD. In reality, this does not happen because of ongoing mortality from all causes.

Two main LOD simulation types are described next:

1. **The individual values analysis of PRSs and risk allele frequency for individuals diagnosed with a disease at each specific age and the remaining population at this age.** The abbreviation “IVA” is used interchangeably with “individual values analysis” in this publication.

The IVA uses 1-year age slices and is performed as follows. Initially, the mean and variance of the PRS for the whole population are calculated. Next, based on the required incidence value for each year, individuals are picked from the unaffected population by randomly sampling the population with a probability proportionate to the individual’s PRS, as summarized in Algorithm 1. These individuals become the cases for the relevant year’s IVA, and the mean and variance of their PRSs are also calculated and recorded. Mortality does not need to be applied to the IVA scenario because it affects the future cases and controls in equal numbers, and accounting for mortality would only result in a smaller population being available for analysis. To track the GWAS statistical discovery power, the same nine representative variants (configurable) are tracked for all LODs simulated. The process continues in this way until the maximum desired simulation age is reached.

1. **A simulated cohort study for each of these diseases.** For the sake of brevity, the word “cohort” is also used throughout this publication.

The clinical study cohort simulation performs an analysis identical to that described above. The difference is that, here, the simulated GWAS clinical studies are performed with a patient age span of 10 years, which is a typical cohort age span, although any age span can be chosen as a simulation parameter. The simulation statistics are collected using the mid-cohort age, which is the arithmetic half-age of the cohort age span. In the first simulation year, a population equal to one-tenth of the complete population goes through the steps described for IVA. Each year, an additional one-tenth starts at age 0, while the previously added individuals age by 1 year. This continues until all 10 ages are represented. This combined cohort proceeds to age and is subject to the disease incidence rate and mortality according to each individual’s age.

Mortality is applied, with a probability appropriate to each year of age, to both accumulated cases and controls. As the population ages, both the case and control pool numbers diminish. Take, for example, a cohort study that includes a 10-year span, say, between 50 and 59 years of age. The cases for the cohort are composed of individuals who were diagnosed with an LOD at any age either younger than or including their current age, producing a cumulative disease incidence over all preceding years of age. For example, some of the individuals that are cases now, at age 59, may have been healthy at age 58. Some of the controls in our cohort at the age of 51 may or may not be diagnosed at an older age, which would qualify them as cases for this cohort, but they are currently younger and healthy. Therefore, it can not be known with certainty the extent to which younger controls differ from cases, except for the fact that they are not currently diagnosed—not unlike a real statistical study cohort. As a result, the corresponding GWAS discovery power can be expected not to change as dramatically as it does for the IVA.

The following additional mortality scenarios were also simulated: (a) double mortality for cases compared with the unaffected population, (b) no mortality for either cases or controls, and (c) a 1-year age span cohort with no mortality for either cases or controls.

The youngest age cohort for each LOD is defined as the mid-cohort age at which the cumulative incidence for a cohort first reaches 0.25% of the population. For consistency, this threshold was considered in this study as the minimum cumulative incidence age, allowing for the formation of well-powered cohort studies for all analyzed LODs.

### The simulation design summary

Preliminary data collection and analysis steps are shared by all simulation runs and include: (a) preparing the genetic architecture files and calculating the number of variants needed, based on each modeled LOD heritability, as described in the Section “Allele Distribution Models” above, and (b) determining the parameters of functional approximation for LOD incidence from published statistics, as described in Chapter S3 in Article S1.

A simulation run for a single LOD can be logically divided into the following four steps:

1. Build the gene variants pool as outlined in the Section “Allele Distribution Models” and load the incidence rate functional approximation parameters.
2. Allocate population objects and assign individual PRSs. Allocate all other simulation objects and arrays that will be used by the simulation.
3. Run the simulation’s Algorithm 1 from age 0 to 100 for either the IVA or the cohort study scenario, described in the Section “Individual Values Analysis and Cohort Simulation.” Calculate and record the simulation data in comma separated value files.
4. Determine statistical power for cases and controls for each cohort based on the Section “Evaluating GWAS Statistical Power.”

The above steps were completely reinitialized and performed for each LOD analysis. The complete simulation iterated through all eight LOD analyses in two scenarios: a per-year-of-age population IVA and a simulated GWAS cohort study.

### Validation simulations

Based on the model described above, it can be expected that the allele distribution in a population of the same age with a given initial genetic architecture will depend solely on cumulative incidence, which represents the fraction of the population that succumbs to a disease. The purpose of validation simulation runs performed with (a) constant, (b) linear, and (c) exponential incidence rates was to validate whether or to what extent this expectation is correct and whether the outcomes would differ between various genetic architectures. The validation simulations confirmed that PRSs for the population controls and cases, viewed in the IVA at every age, depend on the cumulative incidence and the LOD heritability, and are independent from the incidence progression shape within each genetic architecture. The procedures used in the validation simulations are described in Chapter S2 in Article S1.

### Data sources, programming, and equipment

The population mortality statistics from the US Social Security Actuarial Life Table (2014) provided yearly death probability and survivor numbers up to 119 years of age for both men and women.

Disease incidence data from the following sources were extensively used for analysis, using the materials referenced in Chapter S1 in Article S1 for corroboration: AD: (Brookmeyer, Gray & Kawas, 1998; Edland et al., 2002; Kokmen, Chandra & Schoenberg, 1988; Hebert et al., 1995); T2D: (Boehme et al., 2015); CAD and cerebral stroke: (Rothwell et al., 2005); and cancers: (Cancer Statistics for the UK, 2018; Kuchenbaecker et al., 2017).

The simulations were performed on an Intel i9-7900X CPU-based 10-core computer system with 64GB of RAM and an Intel Xeon Gold 6154 CPU-based 36-core computer system with 288GB of RAM. The simulation is written in C++ and can be found in File S1. The simulations used population pools of two billion individuals for the LOD simulations and 300 million for validation simulations, resulting in minimal variability in the results between runs.

The cohort simulations were built sampling at least five million cases and five million controls from the surviving portion of the initial two billion simulated individuals, which is equivalent to 0.25% of the initial population. This means that the cohort study would begin its analysis only when this cumulative incidence was reached. Conversely, the analysis would cease when, due to mortality, the number of available cases or controls declined below this threshold. For all LODs, this maximum mid-cohort age was at least 100 years and, depending on LOD, up to a few years higher. This confirms that, as described later in the Section “Discussion,” in cohorts composed of younger cases and older controls it is feasible to form control cohorts up to 100 years of age.

The simulation runs for either all validation scenarios or for a single scenario for all eight LODs took between 12 and 24 h to complete. The final simulation data, additional plots and elucidation, source code, and the Windows executable are available in Supporting Information. Intel Parallel Studio XE was used for multi-threading support and Boost C++ library for faster statistical functions; the executable may be built and can function without these two libraries, with a corresponding slowdown in execution. The ongoing simulation results were saved in comma separated files and further processed with R scripts during subsequent analysis, also available in File S1.

### Statistical analysis

Large variations between simulation runs complicate the analysis of population and genome models. This issue was addressed in this study by using a large test population, resulting in negligible variability between runs. The statistical power estimates deviated less than 1% in a two-sigma (95%) confidence interval, except for the early AD cohort, which commenced at 1.5% and fell below the 1% threshold within 4 years (see *TwoSDFraction.csv files in File S1). In addition to ensuring that the simulations operated with reliable data, this eliminated the need for the confidence intervals in the graphical display.

## Results

### Validation simulations for all genetic architectures

The validation simulations for all scenarios described in Methods Table 1 were performed not as models of specific diseases but to determine the behavior of all allele scenarios and the resulting allele frequency changes under simple controlled and comparable-to-each-other incidence scenarios. It was important to characterize all genetic architectures and to identify the differences and similarities in behavior between them.

These simulations confirmed that a change in the population’s mean PRS and a change in the cases’ mean PRS, viewed as instantaneous values for each age, are dependent on the cumulative incidence and the magnitude of the initial genetic model heritability. If mortality is excluded, they are not dependent on the shape of incidence progression with age (see Fig. S2 in Article S1) and are qualitatively similar between the genetic architectures (see Fig. S3 in Article S1). This means that, when the same level of cumulative incidence is reached with any incidence pattern, the allele distribution for the diagnosed cases and the remaining unaffected population is identical.

### Analysis of common, low-effect-size genetic architecture scenario

The simulation results for the eight analyzed LODs are presented next.

The IVA and cohort simulations were performed for all genetic architecture scenarios, from low to high effect sizes, and common to low allele frequencies, and the results were found to be qualitatively consistent between all these scenarios. As a consequence, this report primarily focuses on the common low-effect-size genetic architecture scenario A, which the latest scientific consensus considers to be the genetic architecture behind the majority of polygenic LODs; the results are virtually identical for scenarios B and C, as validated in Fig. S3 in Article S1, making it unnecessary to present separate figures for these two scenarios.

**Figure 1:**
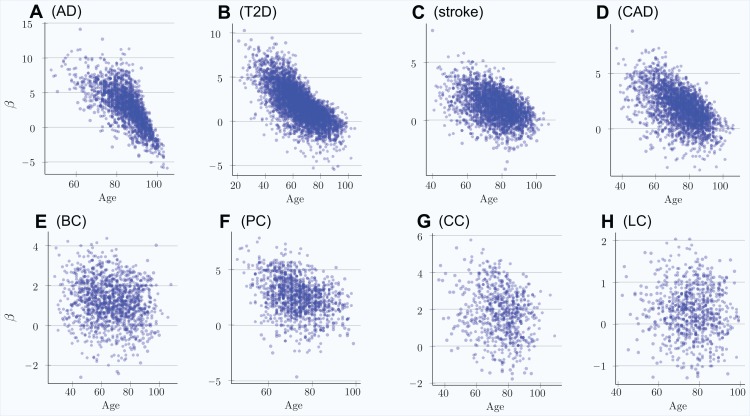
Polygenic risk scores of individuals diagnosed with an LOD as a function of age. (A) Alzheimer’s disease, (B) type 2 diabetes, (C) cerebral stroke, (D) coronary artery disease, (E) breast cancer, (F) prostate cancer, (G) colorectal cancer, (H) lung cancer. Scatter plots show the distributions of PRS for cases diagnosed as age progresses, with ongoing mortality. *Beta* = log(OddsRatio) visually implies the underlying heritability and incidence magnitudes. If the regression line can be easily drawn, dropping diagonally as age progresses, there is a combination of high heritability and increasing cumulative incidence. Otherwise, a plot appears as a relatively symmetrical blob.

**Figure 2:**
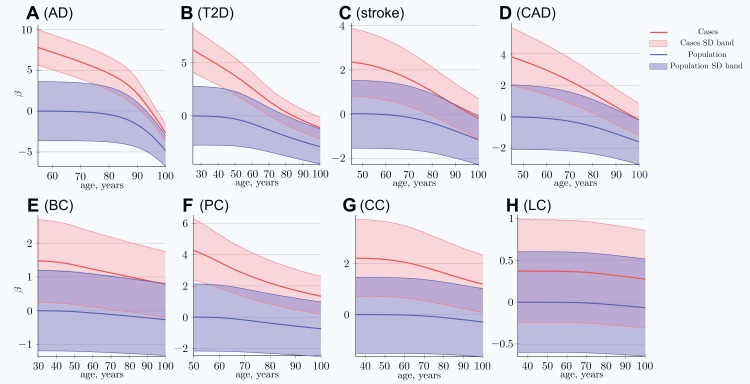
Polygenic risk score difference between newly diagnosed individuals and the remaining unaffected population. (A) Alzheimer’s disease, (B) type 2 diabetes, (C) cerebral stroke, (D) coronary artery disease, (E) breast cancer, (F) prostate cancer, (G) colorectal cancer, (H) lung cancer. *SD band* is a band of one standard deviation above and below the cases and the unaffected population of the same age. For highly prevalent LODs, at very old age, the mean polygenic risk of new cases crosses below the risk of an average healthy person at early onset age. *(Common low-effect-size alleles (scenario A), showing largest-effect variant with MAF = 0.5, OR = 1.15)*.

The scatter plots in Fig. 1 show the distributions of PRS for cases diagnosed as age progresses for the common, low-effect-size genetic architecture scenario A. The PRSs of individuals diagnosed with an LOD and the age-related change of the average LOD PRS of the unaffected population are demonstrated in Fig. 2. The color bands show a one standard deviation spread for cases and controls, which, in the case of newly diagnosed cases, represents approximately two-thirds of the diagnoses at each age. This figure demonstrates how the initially high average polygenic risk of newly diagnosed cases declines as the most predisposed individuals are diagnosed with each passing year of age. The average PRS of the unaffected population decreases much more slowly.

At advanced old age, the average polygenic risk of the newly diagnosed is lower than the risk for an average individual in the population at a young age; this is true for all four highly prevalent LODs: AD, T2D, CAD, and stroke.

**Figure 3:**
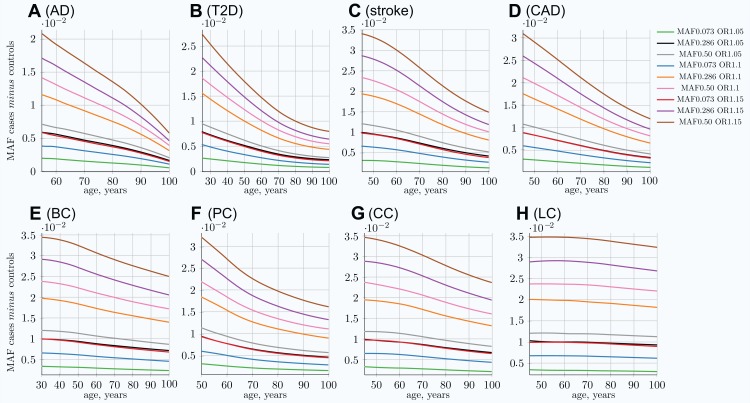
Allele frequency difference between newly diagnosed individuals and remaining population of the same age. (A) Alzheimer’s disease, (B) type 2 diabetes, (C) cerebral stroke, (D) coronary artery disease, (E) breast cancer, (F) prostate cancer, (G) colorectal cancer, (H) lung cancer. The MAF cases *minus* controls value is used to determine GWAS statistical power; see Eq. (6). Rarer and lower-effect-size (OR) alleles are characterized by a lower MAF relative change, see also Fig S5 in Article S1. *(Displayed here: nine out of 25 SNPs, which are described in Methods for common low-effect-size alleles—scenario A)*.

This phenomenon is a consequence of the EAF change, in which the highest-effect alleles show the greatest difference between the diagnosed and the remaining unaffected population as well as the fastest change in frequency difference with age. Statistically, individuals possessing the higher-risk alleles are more likely to succumb and to be diagnosed earlier, thus removing the allele-representative individuals from the unaffected population pool; see Fig. 3. These plots show the most dramatic change for AD and T2D—the LODs with the highest cumulative incidence and heritability. The smallest change corresponds to the LOD with the lowest incidence and heritability: lung cancer.

It is important to note that the absolute MAF values for cases diagnosed at a particular age and controls do not change much with age progression for all LODs. For example, for T2D, the allele frequency for the allele with an OR of 1.15 and an initial population MAF of 0.2860 is 0.2860 for controls and 0.3088 for cases at the age of 25. This changes to 0.2789 for controls and 0.2871 for cases at the age of 80 in the IVA case—a change of only a few percentage points. At the same time, the relative differences change correspondingly from 0.0228 to 0.0081, a 2.7 time change, which is very significant for GWAS discovery power, as can be seen in Eq. (6). The absolute MAF change is even less prominent in the cohort scenario, as can be seen in Fig. S15 in Article S1, which shows the same allele. The small change in the absolute value for older age groups makes it difficult to analyze this effect using, for example, GWAS SNP database statistics for different age groups. The effect would be hidden behind interpersonal and populational genetic variability in hundreds and thousands of SNPs, changing their balance slightly with age in the case of the common, low-effect-size genetic scenario. This effect is long established for highly detrimental variants such as the BRCA1/2 gene mutations in the case of breast cancer (Kuchenbaecker et al., 2017) and the APOE e4 allele in the case of AD (Farrer et al., 1997), where these gene variant carriers are known to be present in lower numbers among older undiagnosed individuals.

**Figure 4:**
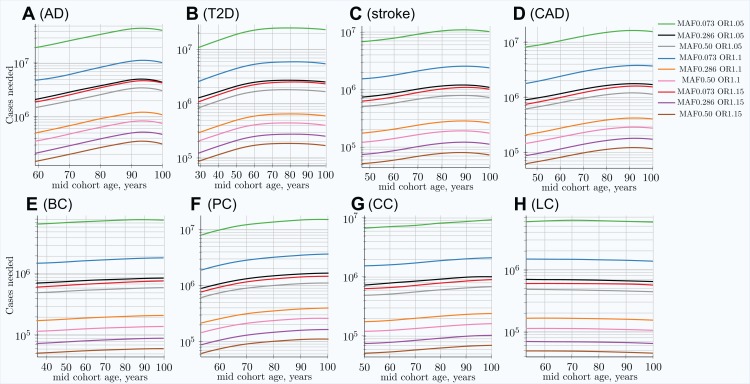
Change in number of cases needed to achieve 80% discovery power in age-matched cases and controls cohort design. (A) Alzheimer’s disease, (B) type 2 diabetes, (C) cerebral stroke, (D) coronary artery disease, (E) breast cancer, (F) prostate cancer, (G) colorectal cancer, (H) lung cancer. Age-matched cohorts require larger numbers of participants to achieve the same GWAS discovery power compared to the youngest cohort age. There is a noticeable difference between cancers (with the exception of prostate cancer; see the Section “Discussion”) and other LODs. *(Displayed here: nine out of 25 SNPs, which are described in the Methods section for common, low-effect-size alleles—scenario A)*.

The cohort simulation shows a much more averaged change for these same scenarios because cohorts represent accumulative disease diagnoses from earlier ages, while mortality removes older individuals; see Fig. S5 in Article S1. While the MAF difference between cases and controls shown in the above figures is illustrative by itself, it is most important for determining the GWAS statistical discovery power using Eqs. (6) and (8) and from there the number of cases necessary to achieve 0.8 (80%) statistical power. From these equations, it is apparent that GWAS statistical discovery power diminishes as a complex function of a square of case/control allele frequency difference. The age-related change in the number of cases needed to achieve 80% GWAS discovery power for an age-matched case/control cohort study is presented in Fig. 4.

In the hypothetical IVA case, the number of individuals required to achieve the desired GWAS discovery power increases rapidly; see Fig. S6 in Article S1. This is a quite informative instantaneous value of statistical power; however, neither GWASs nor clinical studies ever consist of individuals of the same age, due to the need to have a large number of individuals to maximize this same statistical power. The cohort scenario is correspondingly less extreme, as seen in Fig. 4. These plots show an increase in the number of participants needed to achieve adequate GWAS statistical power between the lowest effect and frequency and the highest effect and frequency alleles; this number exhibits a greater-than-hundredfold variation between alleles within the genetic architecture.

**Figure 5:**
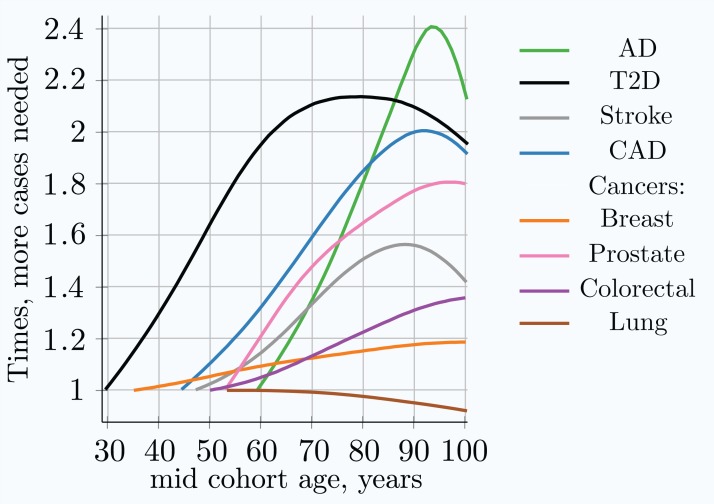
Relative increase in number of cases needed for 80% discovery power in a cohort study using progressively older age matched case and control cohorts. The youngest age cohort for each LOD is defined as the mid-cohort age at which the cumulative incidence for a cohort first reaches 0.25% of the population. Therefore, the leftmost point on each LOD line is the reference (youngest) cohort, and as cohorts age, the cohort case number multiple required to achieve 0.8 statistical power is relative to this earliest cohort. While all alleles display a different magnitude of cases needed to achieve the required statistical power, the change in the multiplier with age is almost identical for all alleles within a given genetic architecture scenario. *(Common low-effect-size alleles scenario A)*.

The required number of cohort participants is quite similar for the same effect alleles among all eight LODs; for example, the highest-effect allele for each LOD requires 5 × 10^4^ − 1.4 × 10^5^ cases for 80% GWAS discovery power at younger ages. The change in allele frequency with age between cases and controls shows substantial variation among LODs, with the greatest change occurring in AD and the least significant in lung cancer; see Fig. 3. Figure 5 summarizes the multiplier—the required increase in the number of participants as the cohort is aging—compared to the youngest possible cohort age for the eight analyzed LODs. These cohort results are simulated with identical mortality for cases and controls. Mortality has an impact on the cohort allele distribution.

Table 4 combines the heritability and incidence of the LODs with the summarized simulation results from the cohort simulation, also seen in Fig. 4 and Fig. S4 in Article S1.

**Table 4:** LOD statistics and age-matched cohort simulation summary.

Disease	Highly prevalent LODs				Cancers			
	AD	T2D	Stroke	CAD	Breast	Prostate	Colorectal	Lung
Lifetime risk %	10–20	55	25–30	32–49	12	12	<4.5	<6.9
Heritability %	79–80	69	38–44	50–60	31	57(42)	40	8–18
Maximum yearly incidence %	>20	2.5	4.4	3.6	<0.5	<0.8	<0.6	<0.6
δMAF between cases and controls								
Youngest cohort	0.020	0.026	0.034	0.032	0.034	0.031	0.034	0.035
Age 80 years	0.015	0.018	0.028	0.023	0.032	0.024	0.031	0.035
Age 100 years	0.014	0.019	0.028	0.023	0.032	0.023	0.029	0.036
Cases needed for 80% statistical power								
Youngest cohort	1.4 × 10^5^	8.7 × 10^4^	5.3 × 10^4^	6.0 × 10^4^	5.0 × 10^4^	6.1 × 10^4^	4.9 × 10^4^	4.9 × 10^4^
Age 80 years	2.6 × 10^5^	1.8 × 10^5^	7.9 × 10^4^	1.1 × 10^5^	5.8 × 10^4^	1.0 × 10^5^	6.1 × 10^4^	4.7 × 10^4^
Age 100 years	3.0 × 10^5^	1.7 × 10^5^	7.3 × 10^4^	1.1 × 10^5^	5.9 × 10^4^	1.1 × 10^5^	6.9 × 10^4^	4.5 × 10^4^
Multiple cases needed, youngest to 80 years	1.9	2.1	1.5	1.8	1.15	1.6 (1.35)	1.25	1.0

**Note:**

The MAF values and cases needed for 0.8 (80%) GWAS statistical discovery power are for the common, low-effect-size alleles, scenario A. Cohorts span 10 years. The results shown are for the allele with a MAF of 0.5 and an OR of 1.15, the largest effect allele, which requires the smallest number of cases/controls. “Maximum incidence %” is the largest incidence at older age. “Case mult.” is the multiple of the number of cases needed for the 80-year-old cohort to achieve the same statistical power as the early cohort. Prostate cancer heritability is 57%, according to Hjelmborg et al. (2014). Shown in braces, 42% heritability (Grönberg, 2003), which is more in line with the other three cancers.

### Validating more extreme mortality scenarios

More extreme mortality scenarios—both lower and higher—than one could expect in a real cohort study were validated in this set of simulations. The results were relatively close to those presented for equal case/control mortality. The extreme cases of (a) no mortality for either cases or controls and (b) double the mortality of cases compared to controls produce very similar allele distributions before the age of 85, while diverging somewhat at older ages. The scenario in which the mortality of cases is double that of controls is higher than the clinically known mortality for the analyzed LODs. While this may have been a realistic scenario a century ago, before modern healthcare, it is certain that patient mortality is lower these days. In addition, a 1-year cohort without mortality was used as the most extreme validation case. This scenario can also be considered an individual cumulative case, which counts everyone who became ill by a specified age as cases and everyone healthy at that age as controls. These validation cohort scenarios are summarized in Fig. S7 in Article S1.

The mortality analysis was applied to one LOD at a time. This research did not attempt to estimate increased mortality for multiple disease diagnoses. Collerton et al. (2009) followed a cohort of individuals over the age of 85 in Newcastle, England, and found that, out of the 18 common old-age diseases they tracked, a man was on average diagnosed with four and a woman with five, not to mention a plethora of other less common diseases and their causal share in individual mortality.

### Evaluating rare, medium-effect-size genetic architecture scenario

Other genetic architecture scenarios produce qualitatively similar patterns, specifically differing in the number of cases needed to achieve 80% statistical power for medium- and large-effect genetic architecture scenarios. The rare, medium-effect-size allele (scenario D) results are presented in Figs. S8–S11 in Article S1. There, at younger ages, the MAF difference between cases and controls is larger for rare, medium-effect-size alleles. The number of cases and controls needed to achieve 80% GWAS statistical power for all eight LODs is approximately five times lower, a direct consequence of these variants’ larger effect sizes. This result perhaps excludes the scenario of rare, medium-effect-size alleles being causally associated with the LODs reviewed here, because GWAS studies would be more readily able to discover a large number of causal SNPs. From a qualitative perspective, all reviewed genetic architecture scenarios provide similar patterns of increasing numbers of cohort study cases needed to maintain the same discovery power with age progression.

## Discussion

Performing a comprehensive set of validation simulations enabled the determination and generalization of the change in allele distribution with an increase in cumulative incidence for all genetic architectures described in the Methods section. The simulation results show that, for all genetic architectures, the change in the PRS depends on the cumulative incidence and the magnitude of heritability. When the same level of cumulative incidence is reached, the difference in allele distribution between diagnosed cases and the remaining unaffected population is identical. It therefore depends on the value of the cumulative incidence, and not on the incidence pattern that led to the achievement of a particular cumulative incidence value in the validation scenarios.

Next, the simulations were performed using the incidence rate patterns and model genetic architectures of each analyzed LOD, determining changes in the allele distribution with age and the resulting impact on GWAS discovery power. These results compared well with the findings from clinical, GWAS, and familial heritability studies, which are summarized below.

There are numerous reports of heritability, clinical predictive power, and GWAS discovery power diminishing with age for these LODs. Almgren et al. (2011) observed T2D heritability equal to 0.69 for the 35–60 year age-of-onset group, and negligible heritability for older ages. GWASs (De Miguel-Yanes et al., 2011; Mühlenbruch et al., 2013) segregating T2D risk SNPs by age have found that the risk factor values are higher for those under the age of 50, compared to the older cohorts. Regarding the variant types that are most likely associated with T2D, Fuchsberger et al. (2016) found that, with a high degree of certainty, they were able to attribute T2D liability to common variants rather than rare, high-effect variants. A cardiovascular disease (myocardial infarction) study by Nielsen et al. (2013) found the predictive power of parental history to decline for ages older than 50. Schulz, Flossmann & Rothwell (2004) found familial history to be the best predictor of ischemic stroke for individuals under the age of 60. A review based on Framingham’s study (Seshadri et al., 2010) found the parental predictive power of stroke to diminish for those aged over 65. The heritability of AD has been estimated at 80% from twin studies (Naj, Schellenberg & Alzheimer’s Disease Genetics Consortium (ADGC), 2017; Gatz et al. (2006) found heritability to be 79% at approximately 65 years of age, diminishing with increasing age. GWASs (Tan et al., 2013; Shen & Jia, 2016; Naj, Schellenberg & Alzheimer’s Disease Genetics Consortium (ADGC), 2017) have come to similar conclusions. In summary, the predictive power of familial history for the above LODs is greatest for younger ages, specifically <65 years of age for AD, <50 for CAD, <60 for stroke, and <50 for T2D.

For the above LODs, the simulation results show high PRSs for the earliest-diagnosed cases. The risk allele case/control difference and the PRSs of newly diagnosed cases decrease rapidly with age progression. At a very old age, the individuals whose genotype would be considered low risk at an earlier age are the ones diagnosed with the disease; see Fig. 2. This also reinforces the validity of the clinical observation that the major risk factor for LODs is age itself.

The four cancers display a noticeably different pattern. The PRSs for the earliest-onset cases are lower than those for the above LODs, and this risk changes much less with age than for the above LODs. These results explain the observations of familial heritability studies: for three out of the four most prevalent cancers, twin studies have shown relatively constant heritability with age progression (Grönberg, 2003; Möller et al., 2016; Mucci et al., 2016; Graff et al., 2017). Determining the change in lung cancer heritability with age has proven somewhat more elusive (Hjelmborg et al., 2016), and no definitive conclusions have been published, largely due to the generally low documented heritability and substantial environmental component of this disease.

Prostate cancer is the only cancer that is somewhat controversial. Its heritability is reported at 57% by Hjelmborg et al. (2014), and prostate cancer reaches the highest maximum instance rate of the four most prevalent cancers reviewed. Therefore, according to the above observations and the results of the validation simulations, the relative MAF between cases and controls is likely to be higher than for other cancers. Nevertheless, the same article finds that the heritability of prostate cancer remains stable with age. It may be that this twin study result is somehow biased and that the heritability of prostate cancer is lower than stated in Hjelmborg et al. (2014), or perhaps this is a phenomenon specific to the populations or environmental effects of Nordic countries. Perhaps the earlier familial study (Grönberg, 2003), which estimated heritability at 42%, would be closer to the UK population incidence data used here. The verification simulation using a heritability of 42% produced results that matched more closely the patterns exhibited by the other cancers; see the resulting value shown in parentheses for the case multiple in Table 4. A more exhaustive literature investigation of the reviewed LODs is presented in Chapter S1 in Article S1.

Genome-wide association studies’ statistical discovery power is impaired by the change in individual distribution of the PRS at older ages. A larger number of cases and controls is needed at older ages to achieve the same statistical discovery power. The first four LODs, which exhibit higher heritability and cumulative incidence compared to cancers, require an increased number of participants in case/control studies for older ages. The cancers show a small increase in the number of participants required to achieve the same statistical power.

Individual values analysis, in which the individuals diagnosed each year are compared to all remaining healthy individuals, shows a rapid increase in the number of cases hypothetically needed to achieve the same statistical power, but this scenario would be impractical for a clinical study. The age-matched cohort studies benefit from the fact that the diagnosed individuals are accumulated from the youngest onset to the age of becoming a case in the cohort study, as well as being averaged over the cohort age range, resulting in a more moderate increase in the number of participants required, or a slower decline in GWAS discovery power for older cohorts. Age-matched cohort studies would require 1.5–2.1 times more participants at age 80 compared to the youngest possible age-matched cohorts in the case of stroke, CAD, AD, and T2D.

## Conclusions

This research was conducted with the goal of establishing whether any of the observational phenomena, including decreasing heritability with age for some notable LODs and the limited success of LOD GWAS discovery, can be explained by changes in the allele proportions between cases and controls due to the higher odds of more-susceptible individuals being diagnosed at an earlier age.

The simulation results reported above show that these phenomena can indeed be explained and predicted by the heritability of the LODs and their cumulative incidence progression. By simulating population age progression under the assumption of relative disease liability remaining proportionate to individual polygenic risk, it was confirmed that individuals with higher risk scores will become ill and be diagnosed proportionately earlier, bringing about a change in the distribution of risk alleles between new cases and the as-yet-unaffected population in every subsequent year of age. With advancing age, the mean polygenic risk of the aging population declines. The fraction of highest-risk individuals diminishes even faster.

While the number of most-susceptible individuals and the mean population susceptibility both decline, the incidence of all LODs initially grows exponentially, doubling in incidence every 5–8.5 years (see the Methods section) and remains high at older ages, leading to a high cumulative incidence for some LODs. The increasing incidence rate in the face of declining polygenic risk for the as-yet-unaffected population can be explained as a consequence of the aging process, which itself is the major risk factor for LODs. In old age, people who have low genetic or familial susceptibility are increasingly becoming ill with an LOD.

Four of the most prevalent LODs—AD, CAD, cerebral stroke, and T2D—exhibit both a high cumulative incidence at older age and high heritability. These simulation results show that a GWAS of any polygenic LOD that displays both high cumulative incidence at older age and high initial familial heritability will be affected by diminishing discovery power when using progressively older age matched cohorts. LODs with low cumulative incidence and low familial heritability produce smaller changes in the allele distribution between affected individuals and the remaining population. As a consequence, the most-prevalent cancers are reported to have stable heritability with age, and therefore these GWASs are less affected by the increasing age of the participant cohorts.

## Supplemental Information

10.7717/peerj.7168/supp-1 Supplemental Information 1 The simulation executable, the source code and the project, R scripts and corresponding batch files for producing functional approximations of clinical incidence and post analysis of simulation results.

10.7717/peerj.7168/supp-2 Supplemental Information 2 Supplemental Article containing three chapters providing literature review and less important methods, and 15 supplemental figures.

## Additional Information and Declarations

### Data Availability

The following information was supplied regarding data availability: Raw data is available in the Supplemental Files.
