# Supplementary material for: Age-related late-onset disease heritability patterns and implications for genome-wide association studies
Source: PeerJ. 2019 Jun 14;7:e7168. doi: 10.7717/peerj.7168 (PMC6573810; doi:10.7717/peerj.7168)
Supplement: Supplemental Information 2 [file peerj-07-7168-s002.pdf]

# Age-related late-onset disease heritability patterns and implications for genome-wide association studies (SUPPLEMENTAL)


## Roman Teo Oliynyk[1,2,*]

[1]Centre for Computational Evolution, University of Auckland, Auckland 1010, New Zealand
[2]Department of Computer Science, University of Auckland, Auckland 1010, New Zealand
[*]roli573@aucklanduni.ac.nz


### Contents:


**1** Supplemental Chapter S1
**2** Supplemental Chapter S2
**3** Supplemental Chapter S3
**4** SUPPLEMENTAL FIGURES


## Supplemental Chapter S1: LOD heritability patterns with age based on familial and clinical studies and genome-wide association studies (GWAS)

The notion that the heritability of LODs always decreases with age is not entirely correct. A review of the clinical and familial studies and GWAS on the heritability of polygenic LODs within the typical age range of disease onset leads to a grouping of LODs into two broad categories: those with decreasing heritability with age and those with increasing or relatively constant heritability with age.

Next, these categories are reviewed in detail, focusing primarily on the eight highly prevalent LODs analyzed in our simulations. These categories are used to organize the observational knowledge to enable the application of this knowledge to the main article's simulations and the verification of the simulation results.

### LODs with decreasing heritability with age

There is a large number of ***highly environmentally affected LODs that exhibit decreasing heritability with age***. Three of these diseases carry some of the highest lifetime risk: coronary artery disease, cerebral stroke, and type 2 diabetes; see Table 1, summarized from Wienke et al. (2001), Zdravkovic et al. (2002), Devan et al. (2013) and Aparicio and Seshadri (2017).

**Supplemental Table 1. Population statistics of LODs characterized by decreasing heritability with age**

| Statistic | Alzheimer's | CAD | Stroke | T2D |
|---|---|---|---|---|
| **Lifetime risk, USA (%)** | 10m, 20w | 49m, 32w | 25m, 30w | 55 |
| **Mortality assigned, USA (%)** | 4.2 | 23.1 | 5.2 | 2.9 |
| **Heritability (%)** | 79 | 50–60 | 38–44 | 69 |
| **Best predictability, age** | <65 | <55 | <60 | <50 |

Lifetime risk numbers, when marked, "w" for women, "m" for men.

Falconer (1967) noted that *"the increase of incidence associated with a variable age of onset can be due to either an increase of the mean liability or an increase of the variance of liability. Consideration of the changes of liability that individuals may undergo as they grow older shows that an increase of variance with increasing age is to be expected, and since the additional variance is likely to be mainly environmental, a reduction of the heritability is to be expected."* Falconer further pointed out that *"the heritability of liability to diabetes, estimated from the sib correlation, decreases with increasing age. For people under 10, heritability is about 70 or 80%, and it drops to about 30 or 40% in people aged 50 and over. The decrease of the heritability is attributable to an increase of environmentally caused variation. The increased environmental variation is not enough to account in full for the increasing incidence, so there is probably also an increase of the mean liability with increasing age."*

In the 1960s, the distinction between autoimmune Mendelian type 1 diabetes and late-onset polygenic type 2 diabetes (T2D) was not known, but it was suspected that there may be two distinct mechanisms. However, this conclusion—of an increase in liability with age, and accordingly blurred heritability—is observed for T2D as well as other LODs.

The greatest heritability for T2D is observed in the 35–60 (0.69) year age of onset group, (Almgren et al., 2011) and heritability declines to only 0.31 when the upper age limit is increased to 75 (making the age range 35–75). In the over-60 group, the "environmental" component is the primary cause of new T2D cases. The environmental component in this case includes systemic and tissue-specific deterioration with age and the cumulative external environmental effects with increased time duration. Just as Falconer did 60 years earlier, this study concludes that T2D heritability decreases with age and that liability may be more accurately predicted in younger individuals.

One review (Talmud et al., 2014) cites two studies that corroborate this view. The first concluded that recalculating the genetic risk for T2D by splitting a cohort by age below and above 50 years using 40 T2D risk SNPs finds that the risk factor values are higher in the younger group (de Miguel-Yanes et al., 2011). Meanwhile, Almgren et al. (2011) correlated the heritability and familiality of T2D with quantitative traits and found a very significant drop in heritability over the age of 60.

The conclusion is that, for reliable GWASs, younger is better: T2D patients under the age of 60—or, even better, under the age of 50—should be chosen. Regarding the variant types that are most likely associated with T2D, Fuchsberger et al. (2016) found that they were able, with a high degree of certainty, to attribute T2D liability to common variants rather than rare, high-effect variants.

Nielsen et al. (2013) cardiovascular disease (myocardial infarction) study provides implicit confirmation of decreasing heritability with age. The predictive power of parental history is as follows: paternal relative risk (RR) = 3.30 for ages <50 and 1.83 for ages >50; maternal RR = 3.23 for ages <50 and 2.31 for ages >50.

Schulz et al. (2004) found familial history to be the best predictor of **ischemic stroke** for individuals under the age of 60, with an overall odds ratio (OR) of 1.73. Relative OR, compared to the under-60 cohort, was 0.95 for the 60–70 age band and 0.77 for individuals over the age of 75.

A review based on Framingham's study (Seshadri et al., 2010) supplies very useful information about parental history of stroke. Even though the grouping used on the parental side is stroke under 65, on the descendant side, there are statistics showing RR both below and above the age of 65. For descendants whose parents had a stroke before the age of 65, the stroke RR was determined. Overall, the RR was 3.79 under the age of 65 and 2.21 over the age of 65; the HR for ischemic stroke was 5.45 under the age of 65 and 2.47 over the age of 65. Additional implicit information from this data, which supports the same conclusion, is listed in Allport et al. (2016)

The heritability patterns for these diseases are summarized in Table 3. There is qualitative and, increasingly, quantitative knowledge about the progressively declining heritability of these diseases at ages above 50, as well as the decreasing associated familial and GWAS predictive power; see Nielsen et al. (2013), Schulz et al. (2004), Seshadri et al. (2010), Bevan et al. (2012), Devan et al. (2013) and Fuchsberger et al. (2016) These studies found familial history to be the better predictor of next-generation disease only when the participants in the parental generation are relatively young; see de Miguel-Yanes et al. (2011), Talmud et al. (2014), Almgren et al. (2011) and Table 1.

An environmental effect on the heritability of cardiovascular disease and T2D with age is evident, (Falconer, 1967; Poulsen et al., 1999) including influences such as spousal environment (Jee et al., 2002).

In addition, T2D is a major co-morbidity factor for CAD and cerebral stroke, as well as causally correlated adiposity and hypertension, which are by themselves associated with CAD and cerebral stroke and other LODs. In the presence of T2D, these diseases develop years and even decades earlier than the typical onset ages (Boehme et al., 2015). For instance, twin studies on the heritability of BMI (a co-morbidity often preceding T2D) show the highest heritability of 85% at 18 years of age, after which heritability slowly declines throughout the lifespan (Elks et al., 2012).

It must be noted that the majority of diseases are influenced to various degrees by environmental factors. The three diseases just reviewed show incomparably higher environmental influence than Alzheimer's disease (AD). For AD, neither lifestyle nor painstakingly developed medications can markedly influence the progression of the disease. In contrast, CAD, cerebral stroke and T2D are often considered by the medical community to be primarily influenced by lifestyle and environment (Lloyd-Jones et al., 2006; Mahmood et al., 2014; Boehme et al., 2015; Diapedia: Epidemiology of type 2 diabetes).

In conclusion, the highly prevalent LODs exhibiting high environmental correlation with onset ages also show decreasing heritability with age. This is combined with an exponential increase in incidence with age. In the case of CAD and cerebral stroke, the exponential incidence rate increase proceeds beyond 80 years of age.

Another type of *LOD showing heritability that declines with age can be described as a mode of failure with aging.* Alzheimer's disease begins relatively late, but from there, its incidence rises exponentially to extremely old age (Brookmeyer et al., 1998). The heritability of Alzheimer's disease is estimated at 80% from twin studies (Naj and Schellenberg, 2017); both familial studies and GWAS estimate heritability at 79% Gatz et al. (2006) at approximately 65 years of age, diminishing with increasing age. Tan et al. (2013); Shen and Jia (2016); Naj and Schellenberg (2017)

A clinical study documenting the association between the APOE genotype and Alzheimer's disease (Farrer et al., 1997; Davidson et al., 2007) reports the change in odds ratio with age of APOE e4/e4 and APOE e3/e4 carriers, which is summarized for the Caucasian population in Table 2.

**Supplemental Table 2. Alzheimer's disease odds ratio by age and APOE alleles, relative to e3/e3 allele carriers**

| *APOE allele* / Age (y) | 55 | 60 | 65 | 70 | 75 | 80 | 85 | 90 |
|---|---|---|---|---|---|---|---|---|
| *e4/e4 OR* | 14.1 | 15.0 | 14.3 | 12.1 | 9.5 | 6.1 | 3.7 | 2.0 |
| *e4/e3 OR* | 3.5 | 3.7 | 3.8 | 3.6 | 3.3 | 2.7 | 2.3 | 1.7 |

Values summarized from Farrer et al. (1997).

Another review (Naj and Schellenberg, 2017) concludes that the typical age at onset is 68.8 years for APOE e4/e4 carriers, 75.5 years for e3/e4 carriers, and 84.3 years for carriers without e4. Moreover, the APOE e4 effect is age dependent, giving a broad-stroke assessment that the e4 allele effect is most prominent between the ages of 60 and 79 and gradually diminishes after the age of 80. This fits well with the assessment (Farrer et al., 1997) summarized in Table 2.

Table 3 summarizes the information in the literature about the decreasing heritability of the LODs referenced above.

The model presented by Brookmeyer et al. (1998) hypothesized that, if the AD incidence curve could be delayed by five years, the overall prevalence of AD would be half the projected rate, assuming unchanged mortality from other causes. AD prevalence in this study is limited by applying

**Supplemental Table 3. Heritability and risk statistics for LODs exhibiting decreasing heritability with age**

| **Disease** | *Heritability/risk, younger age* | *Heritability/risk, older age* |
|---|---|---|
| **AD** e3/e4 (Farrer et al., 1997) | OR=3.8, 65y | OR=1.7, 90y |
| **AD** e4/e4 (Farrer et al., 1997) | OR=15.0, 60y | OR=2.0, 90y |
| **CAD** paternal (Nielsen et al., 2013) | RR=3.30, $< 50y$ | RR=1.83, $> 50y$ |
| **CAD** maternal (Nielsen et al., 2013) | RR=3.23, $< 50y$ | RR=2.31, $> 50y$ |
| **Stroke** (Schulz et al., 2004) | OR=1.63, $< 60y$ | OR=0.77, $> 70y$ |
| **Stroke** all (Seshadri et al., 2010) | RR=3.79, $< 65y$ | RR=2.21, $> 65y$ |
| **Stroke** ischemic (Seshadri et al., 2010) | RR=5.45, $< 65y$ | RR=2.47, $> 65y$ |
| **T2D** (Almgren et al., 2011) | $h^2 = 0.69$, 35-60y | $h^2 = 0.31$, 35-75y |

OR = odds ratio; RR = relative risk; $h^2$ = heritability

a 1.4 mortality multiplier to AD patients compared with the unaffected population.

While AD progression is difficult to influence with lifestyle changes or medications, AD incidence at comparable ages has decreased by about 30% since the 1980s in many Western countries (Binder and Schumacher, 2016; Wu et al., 2017) due to undetermined causes. As life expectancy increases, AD lifetime incidence and prevalence are expected to regain ground.

In conclusion, AD shows an exponentially increasing incidence rate up to the most advanced ages, while also displaying heritability that declines with age.

## LODs exhibiting stable heritability with age

LODs with relatively constant heritability with age and infrequent types of LODs with increasing heritability with age are grouped in this category. As found in the reviewed literature, the increase in heritability, when observed, is moderate. The diseases showing *slightly increasing heritability with age* are found to be those affecting the skeletal system, for instance, osteoarthritis, particularly of large joints such as the hip or lower back. One study (Skousgaard et al., 2015) shows that both the incidence and heritability of advanced osteoarthritis of the hip and lower back increase with age.

It is evident that younger cases are more environmentally and less genetically correlated. For example, osteoarthritis at a younger age is often due to trauma rather than genetics (Amoako and Pujalte, 2014; Warner and Valdes, 2016). At the age of 60, the influence of genetic and environmental components is roughly equivalent, and by the age of 70, heritability increases to 75% and stays close to this level into the 90s. Heritability is even higher and increases with advanced age for osteoarthritis of the spine at multiple locations (Spector and MacGregor, 2004).

The increase in heritability for these diseases is seen to be relatively modest and extends from an initially high level. Many osteoarthritis-affected structures and corresponding diagnoses, with different ages of maximum incidence and heritability by sex and age, do not follow this pattern (Skousgaard et al., 2016).

The osteoporosis findings are similarly varied, with studies finding no heritability of pathology for some bone structures and strong heritability for others (Ralston and Uitterlinden, 2010). Specifically, the osteoporosis associated with bone breaks is very heritable and shows a slight increase in heritability into older age (Shaffer et al., 2008). This is explicable by the fact that, for osteoporosis, the main risk component—the shape and size of the bone—is strongly heritable. Genetics in this case determines the early developmental stages of an organism, when the structures take shape. Similar reasoning applies to osteoarthritis, which is related to defects in collagen and connective tissue formation. The malignancy occurs after many decades of life, when wear, deterioration and diminishing repair capacity cross the threshold leading to pathology.

In conclusion, some LODs with their roots in the early development of an organism's structures may display strong heritability late in life and even increasing diagnostic heritability as aging progresses. GWAS has found only a small set of SNPs that provides very limited risk prediction for these diseases (Loughlin, 2015; Warner and Valdes, 2016). Apparently, the research cannot be impeded by the increasing heritability with age of the GWAS cohorts.

*Relatively stable heritability with advancing age is a distinguishing feature of cancers*. Accurate information about heritability at different ages is not sufficiently explored for most cancers. Fortunately, during this decade, a number of studies have shed light on the age-related heritability of three out of the four most prevalent cancers, and these data allow us to extrapolate the expectations to the fourth: lung cancer.

The lifetime risk of developing any type of cancer in the US is 38% for women and 40% for men, (Lifetime Risk of Developing or Dying From Cancer) and the 2016 fraction of mortality directly attributed to cancer was 21.8%, the second-highest after heart disease (Murphy et al., 2017). In the UK, the corresponding numbers are higher, at 47% and 53%, respectively, (Ahmad et al., 2015; Cancer Statistics for the UK) with the higher likelihood perhaps attributable to the UK's longer life expectancy. Each specific type of cancer constitutes a small fraction of overall lifetime risk, with breast, prostate, lung, and colorectal cancer being the four most prevalent.

Next, the latest heritability and incidence research for these four cancers is summarized.

**Breast cancer (BC)** Breast cancer (BC) is well researched, with studies delving into all aspects of BC. Like prostate cancer, the two largest genetic predictors of BC are mutations in the BRCA1 and BRCA2 genes. The BRCA1/2 genes are involved in the homologous repair of double-stranded DNA breaks, working in combination with at least 13 known tumor suppressor proteins (Haley, 2016). Defects in the BRCA1/2 proteins disable homologous double-stranded DNA break repair, and the cell falls back on the use of imprecise non-homologous repair mechanisms; this leads to the accumulation of mutations, eventually leading to cancer. BRCA1/2 mutations are the most important predictor of breast cancer. The review by Haley (2016) states that the frequency of BRCA mutations varies with geographic location and ethnicity, ranging from a 0.02% mutation carrier rate in some populations to 2.6% in the Ashkenazi Jewish population due to ancient founder mutations. Other founder mutations have been reported in the Dutch, Swedish, French Canadian, Icelandic, German, and Spanish populations. In On-

tario, Canada, for instance, the frequency of mutation carriers is 0.32% for BRCA1 and 0.69% for BRCA2 (Risch et al., 2006).

An early study (Ford et al., 1998) analyzing families with at least four cases of BC found that the disease was linked to BRCA1 in 52% of cases and BRCA2 in 32% of cases (with only 16% remaining for other causes). Taking into account ovarian cancer in addition to BC resulted in 81% of cases being due to BRCA1, while 76% of cases in families with both male and female BC were due to BRCA2.

The lifetime risk of BC for women both in the US and the UK is 12% (Lifetime Risk of Developing or Dying From Cancer; Cancer Statistics for the UK). As Haley (2016) summarized, carriers of BRCA1 have a lifetime risk of developing BC equal to 60–70%, and an additional 40% risk of developing ovarian, fallopian, or primary peritoneal cancers. For BRCA2 carriers, the risks are 45–55% for BC and 25% for ovarian cancer. These numbers closely correspond to the aforementioned study (Ford et al., 1998).

Möller et al. (2016) presented in-depth data on the heritability by age of breast and ovarian cancer for BRCA1/2 carriers. The study demonstrated that the genetic liability, while exhibiting a slight downward trend, remains relatively constant and exceeds the common environmental component at all ages.

One of the most recent studies (Kuchenbaecker et al., 2017) provides further clarification, stating that BC incidences increase rapidly in early adulthood until the ages of 30 to 40 for BRCA1 carriers and until the ages of 40 to 50 for BRCA2 carriers, thereafter remaining at a relatively constant incidence rate of 2–3% per year until at least 80 years of age; see Table 4. This study's calculations based on this data show that the initial increase in incidence is exponential before flattening into the constant horizontal incidence rate approximation; a logistic approximation also fits. The exponential doubling rate, until it reaches the constant incidence level, is also consistent with all other diseases reviewed, showing an incidence doubling time of five years for BRCA1 and eight years for BRCA2 (the BRCA1 calculation, based only on two data points, is less accurate). A much earlier review study (Antoniou et al., 2003) collected the same kind of statistics as Kuchenbaecker et al. (2017) and arrived at similar conclusions.

**Supplemental Table 4. BRCA1/2 carriers incidence rate by age, data from Kuchenbaecker et al. (2017)**

| Gene | ≤20 | 21–30 | 31–40 | 41–50 | 51–60 | 61–70 | 71–80 |
|---|---|---|---|---|---|---|---|
| BRCA1 (%) | 0 | 0.59 | 2.35 | 2.83 | 2.57 | 2.50 | 1.65 |
| BRCA2 (%) | 0 | 0.48 | 1.08 | 2.75 | 3.06 | 2.29 | 2.19 |
| BRCA1 cum. risk (%) | 0 | 4 | 24 | 43 | 56 | 66 | 72 |
| BRCA2 cum. risk (%) | 0 | 4 | 13 | 35 | 53 | 61 | 69 |

Möller et al. (2016) study found a somewhat lower lifetime BC risk of 8.1% in Nordic countries compared to 12% in the US and estimated heritability at 31%.

In addition to BRCA1/2, Mavaddat et al. (2010) and Haley (2016) also list a number of high-penetrance gene mutations—the TP53, PTEN, STK11, and CDH-1 gene mutations—giving a lifetime probability of cancers in general of about 90% and specifically a female breast cancer probability above 50%.

Several rare gene mutations—CHEK2, PALB2, ATM, BRIP1CHEK2, PALB2, ATM, and BRIP1—are also associated with a breast cancer relative risk in the range of 1.5–5.0. In aggregate, these high-effect mutations are correlated with only approximately 10% of hereditary breast cancers (Risch et al., 2006; Haley, 2016).

To date, GWAS attempts to discover common polygenic variants of low effect size have had only limited success. One review study (Lyra-Junior et al., 2017) outlines the history and accomplishments of breast cancer GWAS over a decade of research. The most recent high-powered consortium study (Michailidou et al., 2017) included 122,977 cases and 105,974 controls of European ancestry as well as 14,068 cases and 13,104 controls of East Asian ancestry. The study verified 102 previously reported SNPs, finding that 49 of them were reproducible. The study also found that the majority of discovered SNPs reside in non-coding areas of the genome. The discovered set of polygenic SNPs allows for the explanation of approximately 4% of heritability on top of the 14% explained by known high-penetrance SNPs, bringing the predictive power to 18%. This GWAS estimated the familial heritability of breast cancer at 41%—a possible exaggeration, because it significantly exceeds the 31% estimated by Möller et al. (2016) and the 27% estimated by Mucci et al. (2016)

***Breast cancer conclusions:*** The familial heritability studies and BRCA1/2 clinical studies show that breast cancer heritability is relatively constant over the age of 40 for both mutations. A number of high-penetrance gene mutations can explain an additional fraction of heritability, totaling 10–14%.

The GWAS described above (Michailidou et al., 2017) also found multiple SNPs located in non-coding areas to be correlated with the candidate gene promoters and activity modifier areas. This improves the possibility that the common variant component may be able to explain a larger fraction of heritability. It appears at this time, based on Möller et al. (2016) statistics, that breast cancer heritability for the polygenic component may also be relatively constant after the age of 40 or may only slightly decline with age.

**Prostate cancer (PC)** The effects and risks of the BRCA1/2 genes and their mutations described in the breast cancer section apply in a very similar way to the incidence of PC.

A study by Lecarpentier et al. (2017) found that lifetime PC risks are approximately 20% for BRCA1 mutations carriers and 40% for BRCA2 mutation carriers, while, overall, BRCA1/2 is associated with only 2% of all PC cases. In addition, BRCA1/2 accounts for 10% of male breast cancer cases. The lifetime risk of male breast cancer in mutation carriers is estimated at 5–10% for BRCA1 mutations and 1–5% for BRCA2 mutation carriers. Therefore, compared to breast cancer, BRCA1/2 mutations are associated with a smaller fraction of heritability.

The lifetime risk of PC in men is estimated at 6% for Danish cohorts and 12% for Finnish, Norwegian, and Swedish cohorts. The lifetime risk of developing PC in the US and

the UK is 12% (Lifetime Risk of Developing or Dying From Cancer; Cancer Statistics for the UK). PC heritability has been estimated at 57% (Hjelmborg et al., 2014; Mucci et al., 2016) and 42% by an older study (Grönberg, 2003). The Nordic twin study (Hjelmborg et al., 2014) presents strong evidence that the heritability of PC remains stable or even slightly increases between the ages of 65 and 100. As with breast cancer, the fraction of PC attributed to highly malignant mutations is low. Known rare, high-effect-size variants such as BRCA1/2, ATM, and HOXB13 explain only 10–12% of heritability (Wu and Gu, 2016; Mancuso et al., 2016; Walsh, 2017; Lecarpentier et al., 2017). Recently, Eeles et al. (2017) using an imputed meta-analysis for 145,000 men, reported that the GWAS polygenic score they obtained explains 33% of the familial relative risk.

Wu and Gu (2016) concluded that the search for the missing heritability may be better served by high-coverage whole-genome sequencing (WGS); however, due to the cost and complexity, it is not currently feasible to obtain this much high-quality data. In the absence of more predictive genetic data, Wu and Gu (2016) noted that the best predictor of PC is age itself.

***Prostate cancer conclusions:*** The conclusions for PC heritability are very much the same as for breast cancer. While the heritability is higher than that of BC, it appears even more likely to remain constant or slightly increase with age, notwithstanding the smaller number of known rare, large-effect-size mutations that can be used to explain the heritability of PC.

**Colorectal cancer (CRC)** The lifetime risk of developing CRC in the US is 4.1% for women and 4.5% for men (Lifetime Risk of Developing or Dying From Cancer). In the UK, the corresponding numbers are 5% and 7% (Cancer Statistics for the UK).

The Nordic twin studies (Mucci et al., 2016; Graff et al., 2017) estimated CRC heritability at 40%. A number of studies have included separate classifications for colon cancer, with a heritability of 15%, and rectal cancer, with a heritability of 14%, while the combined percentage is more than double the individual ones. This example may indicate that, while subdivisions exist in the medical diagnoses that may make a difference for surgical or treatment purposes, and while even the carcinogenicity manifestations may differ between subareas of the organ, from the perspective of the heritability of the liability, they are inherited as a single condition.

CRC heritability is also relatively constant between the ages of 50 and 95 in twin studies (Graff et al., 2017). Compared to the two previously reviewed cancers, there is a larger number of identified predisposing mutations and syndromes, such as Lynch syndrome, familial adenomatous polyposis, Peutz–Jeghers syndrome, juvenile polyposis syndrome, MUTYH-associated polyposis, NTHL1-associated polyposis, and polymerase proofreading-associated polyposis syndrome (de Voer et al., 2016; Jiao et al., 2014).

Graff et al. (2017) study concluded that, although a small number of genetic variants have a substantial effect on CRC, a considerable portion of its heritability is thought to result from multiple low-risk variants. de Voer et al. (2016)) concurred that penetrant high-effect gene variants are found in 5–10% of CRC cases. A GWAS review (Schmit et al., 2016) found that more than 50 SNPs have been identified as credibly associated with CRC risk, yet these only account for a small proportion of heritability. In GWAS, common, genome-wide variants are able to account for 8% of heritability.

***Colorectal cancer conclusions:*** The conclusions are much the same as for BC and PC.

**Lung cancer (LC)** The lifetime risk of developing LC in the US is 6.0% for women and 6.9% for men (Lifetime Risk of Developing or Dying From Cancer). In the UK, the corresponding numbers are 5.9% and 7.6% (Cancer Statistics for the UK).

The LC pattern of heritability is not easy to ascertain. According to Kanwal et al. (2017) approximately 8% of lung cancers are inherited or occur as a result of a genetic predisposition. The Nordic twin studies review (Mucci et al., 2016) estimated the heritability of LC at 18% (within a likely range of 0–42%). Heritability studies require controlling for environmental factors, particularly tobacco smoking. It is perhaps for this reason that the Nordic twin studies consortium, which was invaluable in the three other cancer analyses, primarily restricted itself to analyzing the effects of tobacco smoking on LC (Hjelmborg et al., 2016).

Factors such as asbestos, industrial smoke and pollutants, high levels of domestic radon in some areas of the world, or exposure of miners to radon or other sources of radiation may influence incidence and, if not accounted for, may affect heritability estimates (Krewski et al., 2005; Carr et al., 2015; Malhotra et al., 2016). Hereditary mutations of genes that regulate DNA repair, including BRCA1/2, TP53 and others, also increase the risk of LC, as with almost any cancer (Kanwal et al., 2017).

Due to the low heritability of LC, GWASs' success at identifying predictive common SNPs has been limited (Weissfeld et al., 2015). Some studies explain part of the LC incidence by reference to causal epigenetic effects (Shi et al., 2017). The heritability value of 18% given by Mucci et al. (2016) has a very broad range. An earlier study (Yang et al., 2013) noted that tobacco smoking is by far the largest causal factor for LC, and the heritability of smoking itself may outweigh any other LC heritability.

Mucci et al. (2016) also considered smoking, but the high value reported by them exceeds the previous consensus and may need further corroboration. LC perhaps belongs to the difficult-to-analyze, non-additive traits of heritability noted by Polderman et al. (2015). This study considers LC heritability to be closer to 10%.

***Lung cancer conclusions:*** In conclusion, an age-related heritability pattern for LC is lacking, and while it is impossible to make definitive conclusions, it can be hypothesized that LC follows a similar pattern to the other three cancers reviewed.

In summary, the heritability patterns of cancers were not systematically investigated until relatively recently. A

small number of familial studies (Hjelmborg et al., 2014; Möller et al., 2016; Haley, 2016; Graff et al., 2017) and a more recent study that is particularly informative about the incidence of BRCA1/2 mutations with age (Kuchenbaecker et al., 2017) have finally allowed researchers to determine that cancer heritability remains relatively constant with age. Table 5 summarizes the findings from the reviewed literature in relation to breast, prostate, colorectal, and lung cancer. Studies ascertaining the heritability of lung cancer with age are absent from the literature; data may be difficult to collect due to the relatively low heritability of the disease.

Most lung cancer incidence is environmental, and lung cancer does not have specific, highly malignant mutations that may cause a noticeable fraction of heritability. The mostly polygenic fraction of lung cancer heritability is hypothesized to be similarly stable with age, as is the case with the other three cancers reviewed.

**Supplemental Table 5. Patterns of heritability by age for most common cancers**

| Cancer type: | Breast | Prostate | Colorectal | Lung |
|---|---|---|---|---|
| Lifetime risk, USA (%) | 12 | 12 | 4.5m 4.1w | 6.9m 6w |
| Heritability (%) | 31 | 57 | 40 | 8–18 |
| Incidence from highly detrimental mutations (%) | 10–14 | 10–12 | 5–10 | minor |
| Polymorphic incidence (%) | 86–90 | 88–90 | 90–95 | major |
| Heritability trend (50y–100y) | flat / slight decline<br><br>(Ford et al., 1998; Antoniou et al., 2003; Risch et al., 2006; Mavaddat et al., 2010; Mucci et al., 2016; Haley, 2016; Möller et al., 2016; Kuchenbaecker et al., 2017; Michailidou et al., 2017) | flat / slight incline<br><br>(Hjelmborg et al., 2014; Wu and Gu, 2016; Mancuso et al., 2016; Walsh, 2017; Lecarpentier et al., 2017; Eeles et al., 2017) | flat<br><br>(Jiao et al., 2014; Schmit et al., 2016; de Voer et al., 2016; Graff et al., 2017) | likely flat<br><br>(Krewski et al., 2005; Weissfeld et al., 2015; Carr et al., 2015; Malhotra et al., 2016; Mucci et al., 2016; Hjelmborg et al., 2016; Kanwal et al., 2017; Shi et al., 2017; Wang and Wang, 2017) |

Lifetime risk numbers, when marked, "w" for women, "m" for men.
Lifetime risk numbers, when marked, “w" for women, “m" for men.

Because cancer development is primarily a consequence of mutations and epigenetic effects leading to unconstrained propagation of the clonal cell population, in the long term, cancers are inevitable for most multicellular organisms, including humans (Marusyk and DeGregori, 2008; Tomasetti and Vogelstein, 2015; Guedj et al., 2016; Ribezzo et al., 2016; Nelson and Masel, 2017).

Due to cancer's constant heritability with age, the effect of age is likely to be insignificant for GWASs' discovery of cancer polygenic scores and their corresponding predictive power. This could also apply to any LOD that follows a similar heritability pattern, that is, one that is relatively constant with age.

# Supplemental Chapter S2: Incidence functional approximation used in preliminary validations

To determine the effect of disease incidence with age progression on allele frequencies in the population and the difference in allele frequency between the newly affected and remaining unaffected populations, three incidence dependencies with age were used.

1) Constant incidence:

$$I(t) = a, \tag{1}$$

where $a$ is a constant representing a horizontal line. Yearly incidence values of 0.0015, 0.005, and 0.02 (0.15% to 2%) were selected.

2) Linear incidence:

$$I(t) = b\,t, \tag{2}$$

where $b$ is a slope of the linear progression with intercept 0. Slope values of 0.003, 0.01, and 0.04 were selected. This means that incidence begins at 0 and increases to an incidence equal to 0.3%, 1%, and 4%, respectively, at 100 years of age to match the cumulative incidence of 1) above.

These values were chosen to simplify the evaluation via simulation. The simulation was run with zero mortality, and the values were chosen to keep cumulative incidence at the same level—0.44 (44%)—at 100 years of age for the highest of either the constant or linear incidence progression.

3) In addition, an evaluation exponential incidence progression was used:

$$I(t) = 3.05 \cdot 10^{-5} e^{0.1178t}, \tag{3}$$

fitted to achieve a similar cumulative incidence at the most advanced age.

In all five scenarios described in the main article, the values of the case and control means and standard deviation/variance are identical when the cumulative incidence reaches the same level.

Two heritability scenarios were validated: 30.5% and 80.5%; see Table 6.

**Supplemental Table 6. Linear and constant incidence validation scenarios**

| | A | B | C | D | E |
|---|---|---|---|---|---|
| Scenario 1. Variants: | 400 | 625 | 1375 | 50 | 25 |
| Achieved heritability: | 0.3068 | 0.308 | 0.3075 | 0.296 | 0.3142 |
| Scenario 2. Variants: | 3725 | 5850 | 12775 | 500 | 225 |
| Achieved heritability: | 0.8047 | 0.8064 | 0.8049 | 0.8078 | 0.8048 |

The target heritability is 0.305 (30.5%) for validation scenario 1 and 0.805 (80.5%) for validation scenario 2 due to the genetic architecture model requiring multiples of 25 variants.

## Validating allele distribution change in model genetic architectures using systematic incidence progressions

A set of validation simulations was run to verify the behavior of the model genetic distributions for the three types of incidence progression described above. The validation simulations based on the constant, linear and exponential incidence rates confirmed that both of the mean polygenic scores, for the population and for the cases, viewed in the individual values analysis for each age depend on the cumulative incidence and the magnitude of heritability, with neither being dependent on the shape of incidence progression with age.

From the validation simulations, the cumulative incidence, regardless of the incidence progression pattern, was found to produce a virtually identical polygenic score distribution for cases and the remaining unaffected population; see the genetic common allele low effect size plotted in Supplemental Fig. 2.

Between the genetic architectures, there is also a relatively small difference in the polygenic scores of the population and the cases; see Supplemental Fig. 3. As can be seen, the low-effect-size scenarios A, B, and C, progressing in allele frequency from common to rare, are practically indistinguishable from each other.

The higher-effect-size architectures (D and E) show a slightly larger fraction of higher-polygenic-score individuals or, more precisely, a slightly larger representation of higher- and low-polygenic-score individuals. The qualitative picture is close to identical among all five scenarios.

## Supplemental Chapter S3: LOD incidence functional approximation

The simulations were applied to eight of the most prevalent LODs: Alzheimer's disease, type 2 diabetes, coronary artery disease, and cerebral stroke, and four late-onset cancers: breast, prostate, colorectal, and lung cancer.

First, the functional approximation of the clinical incidence data used for the simulations is described. The incidence progression of the LODs with age is presented in Supplemental Fig. 1. The initial incidence rate (the fraction of the population newly diagnosed each year) increases exponentially with age. This exponential growth continues for decades, after which the growth in older cohorts may flatten, as in the case of T2D (Boehme et al., 2015). In the case of cerebral stroke and CAD, the clinical studies indicate a slowdown of the incidence for individuals over the age of 85; (Rothwell et al., 2005) accordingly, a constant level was used for the exponential approximation Eq. 4.

The incidence of Alzheimer's disease, on the other hand, continues exponentially past the age of 95, reaching incidences above 20% (Brookmeyer et al., 1998). Cancer progression reaches only a small fraction of the incidence levels of the above-mentioned LODs, even for the four most prevalent cancers. Generalizing to other cancers, the incidence is much lower for more than a hundred of the less prevalent cancer types.

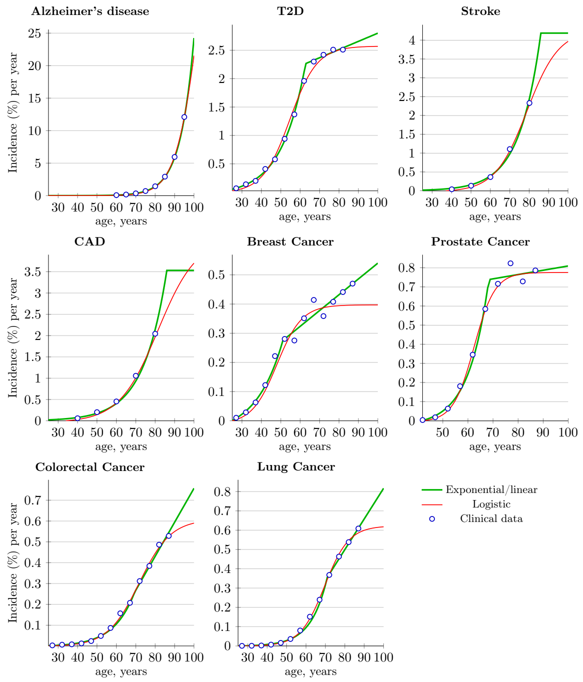


**Supplemental Fig. 1. LOD clinical incidence rates and functional approximations.**

Two functional approximations of clinical data: exponential followed by linear and logistic. The R script automating NLM (nonlinear model) regression for both approximation curves is available in Supporting Information.

To evaluate each LOD's allele redistribution with age, it was necessary to approximate the yearly incidence from much rougher-grained statistics. An R script automated the determination of the best fit for logistic and exponential regression from the clinical incidence data. The script also calculated lifetime incidence from our functional approximations; it closely matched the disease clinical statistics presented in Tables 1 and 5.

The incidence approximation $I(t)$ is represented mathematically by Eq. 4; $a$, $b$, and $c$ are exponential approximation parameters, $i$ and $s$ are the linear regression intercept and slope, respectively, and $t$ is time in years.

$$I(t) = \begin{cases} ae^{bt} + c, & \text{until intersection with the line, below} \\ i + st, & \text{thereafter} \end{cases} \quad (4)$$

A logistic approximation of the clinical data is shown in red in Supplemental Fig. 1. It is characterized by the following equation:

$$I(t) = \frac{a}{1 + e^{(c-t)/b}} + d. \quad (5)$$

The incidence rate in the logistic curve slows faster than the incidence rate in the exponential curve and also approximates the incidence rate with age. It follows a similar pattern, with an initial exponential rise and a logistic inflection point occurring at quite advanced ages. Thus, the clinical data and corresponding approximations show the higher representation of older people in the patient cohorts.

For all LODs, decades-long initial exponential sections were observed in the incidence curve. The exponent conforms to a relatively narrow range of doubling the incidence rate, fitting between 5 and 8.5 years. While the absolute incidence rate differs significantly, the exponent constant multiplier $a$, which is equivalent to the linear regression intercept for $log(a)$ in the $I(t)$ function, mainly controls the rise, or the initial incidence onset, of the incidence rate; see Supplemental Fig. 1.

From this are found the logistic recursion inflection points at values shown in Table 7. The exponential incidence rise follows with high precision up to the ages shown in the table, and the rapid rise in the incidence rate continues past these ages.

**Supplemental Table 7. Age to which LOD incidence rate rises exponentially**

| | Highly prevalent LODs | | | | Cancers | | | |
|---|---|---|---|---|---|---|---|---|
| | AD | T2D | CAD | Stroke | Prostate | Colorectal | Breast | Lung |
| Age (years) | 103 | 55 | 81 | 79 | 48 | 62 | 72 | 70 |

The logistic approximation produced a good, simple fit for seven of the eight diseases. While the logistic approximation could also have been used for breast cancer, the exponential-plus-linear approximation showed a better fit and was therefore preferred.

As this paper makes extensive reference to the incidence of LODs, some of the commonly used terms are clarified below. A lifetime incidence, also called a cumulative rate, is calculated using the accepted method of summing the yearly incidences: (Sasieni et al., 2011)

$$I_{lifetime} = \sum_{t=0}^{t_{max}} I(t), \tag{6}$$

For larger incidence values, the resulting sum produces an exaggerated result. It may become larger than 1 (100%), in which case the use of an approximate clinical statistic called cumulative risk overcomes this issue and is more meaningful. This is much like compound interest, which implicitly assumes an exact exponential incidence progression (Sasieni et al., 2011)

$$CumRisk = 1 - e^{-I_{lifetime}}. \tag{7}$$

Cumulative risk (Eq. 7) is also an approximation because, in any practical setting, the statistic is complicated by ongoing population mortality, multiple diagnoses, and other factors. In addition, cumulative incidence and cumulative risk can be used to find values for any age of interest, not only lifetime. When necessary in this study's simulations, the exact diagnosis counts were used to calculate the precise cumulative incidence for every age.

# SUPPLEMENTAL FIGURES

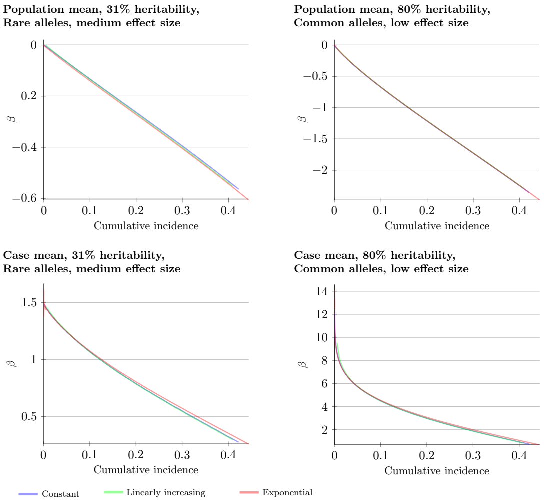


**Supplemental Fig. 2. Validation simulations: constant, linear, and exponential incidence curves within the same allele architectures.**
Using a constant incidence at a level of 0.5% per year, linearly increasing incidence with a slope of 0.01%, and exponentially reaching similar cumulative incidence in a 105-year age interval. Within the same allele architecture, the $\beta$ is identical, subject to the simulation population stochasticity; $\beta = log(OddsRatio)$.

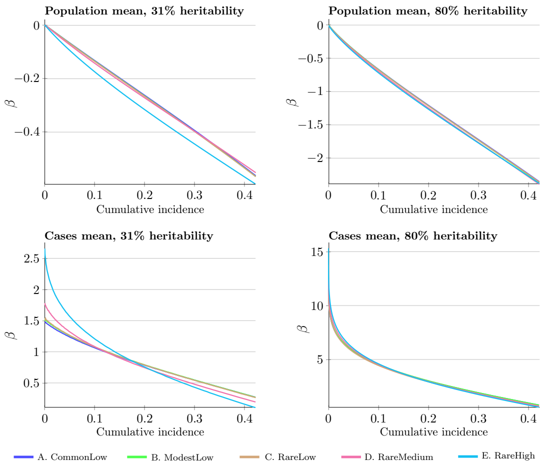


**Supplemental Fig. 3. Validation simulations for five allele architectures.**
The linear and constant incidence patterns give identical results for each allele architecture. The rare medium-effect-size and even rarer high-effect-size scenarios produce a fraction of higher individual betas for the same overall population variance; a relative difference is less prominent at 80% versus 31%. The three identical low-effect-size scenarios produce effectively identical $\beta$ patterns; $\beta = log(OddsRatio)$.

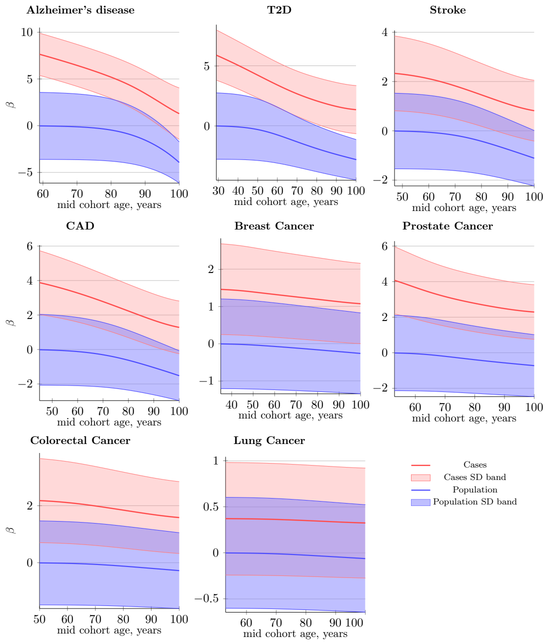


**Supplemental Fig. 4. Polygenic score difference between patients and controls in a cohort simulation.**
Common, low-effect-size alleles (scenario A); $\beta = log(OddsRatio)$. *SD band* is a band of one standard deviation above and below the cases and the unaffected population of the same age. The cohort change and difference are less prominent than in IVA due to the accumulated diagnoses from younger cases with an averaged control polygenic risk score and mortality.

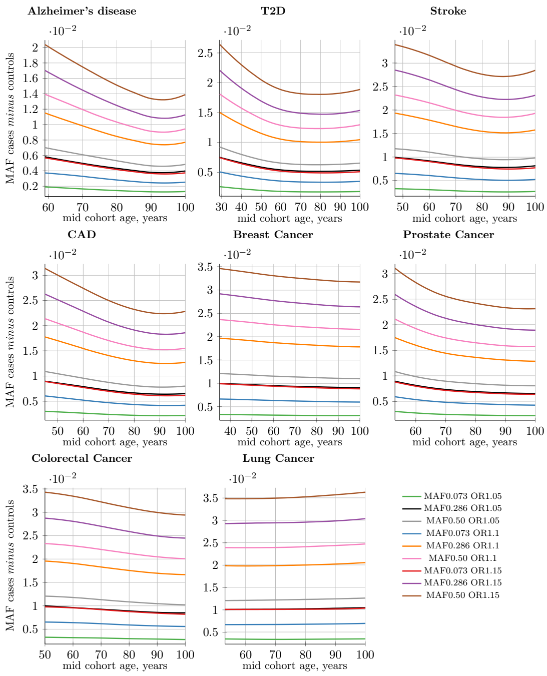


**Supplemental Fig. 5. Allele frequency difference between cases and controls; cohort simulation.**
Common low-effect-size alleles (scenario A). The **MAF cases *minus* controls** value is used to determine GWAS statistical power. Rarer and lower-effect-size (OR) alleles are characterized by a lower relative MAF change.

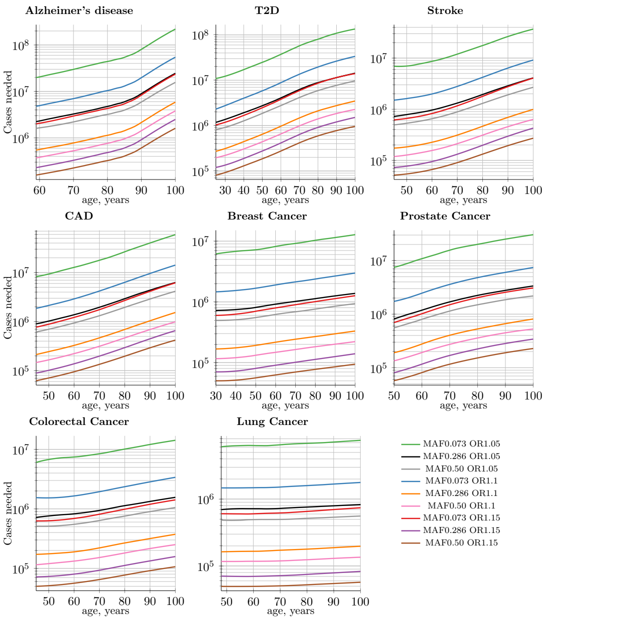


**Supplemental Fig. 6. Number of cases needed to achieve 0.8 discovery power; IVA.**
Common, low-effect-size alleles (scenario A). The diagnosed-individuals-versus-same-age-unaffected-population curve continues to rise steeply in the IVA scenario. A sample of 9 out of 25 SNPs; MAF = minor (risk) allele frequency; OR = risk odds ratio.

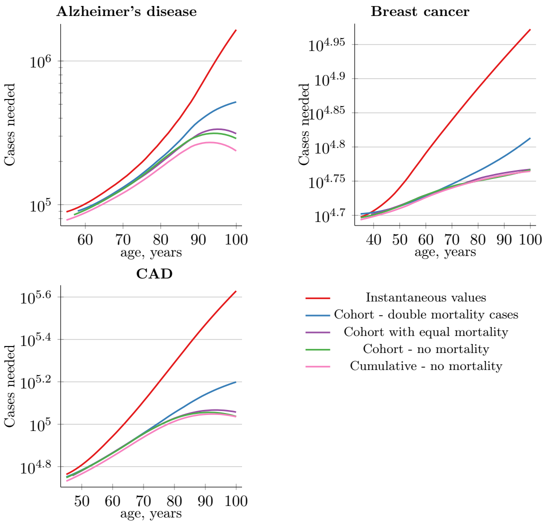


**Supplemental Fig. 7. Number of cases needed for 0.8 discovery power for three LODs with representative incidence rate and initial heritability; summary of five LOD validation simulation types.**
The number of cases needed for 0.8 GWAS discovery power for the clinical cohort study scenario lies between equal mortality for cases and controls and double mortality for cases; it is closer to equal mortality for the LODs we review. The divergence begins after age 85 and is even then relatively modest. "Cohort—double mortality" cases have a mortality twice as large as controls (doubling the value for mortality from the US "Actuarial Life Table". "Cumulative—no mortality" is the most extreme case of a one-year-span GWAS cohort; with no mortality, it requires the smallest number of cases in GWAS. Note that the logarithmic scale is very different among the three LODs.

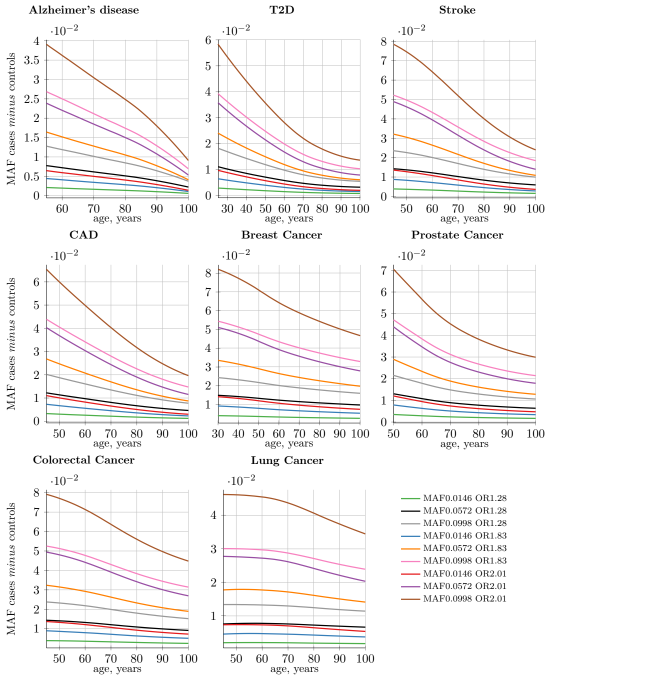


**Supplemental Fig. 8. Difference in allele frequency between newly diagnosed instances and the remaining unaffected population; IVA.**
Rare, medium-effect-size alleles (scenario D). The **MAF cases *minus* controls** value is used to determine GWAS statistical power. Rarer and lower-effect-size (OR) alleles are characterized by a lower relative MAF change.

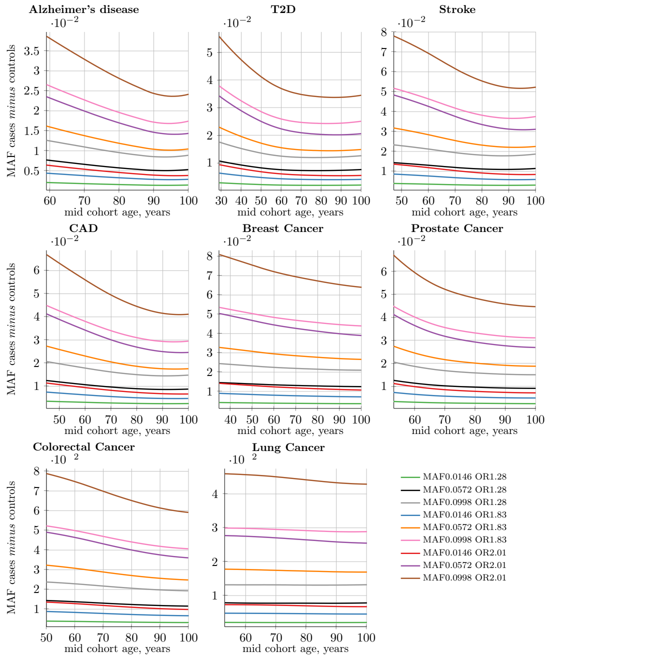


**Supplemental Fig. 9. Difference in allele frequency between cases and controls; cohort simulation.**
Rare, medium-effect-size alleles (scenario D). The **MAF cases *minus* controls** value is used to determine GWAS statistical power. Rarer and lower-effect-size (OR) alleles are characterized by a lower relative MAF change.

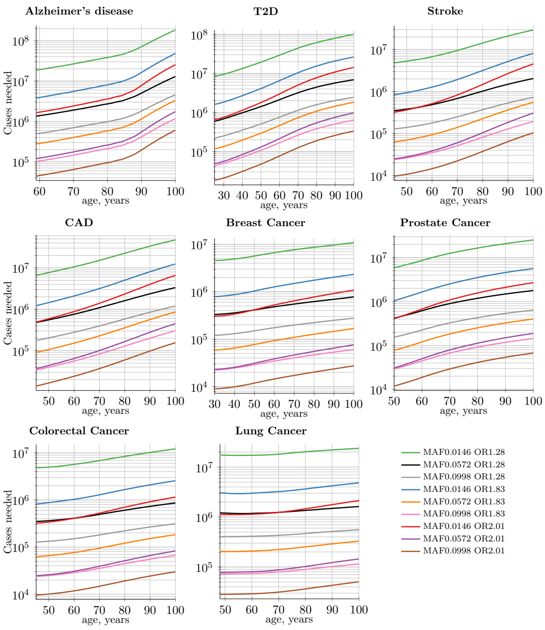


**Supplemental Fig. 10. Number of cases needed to achieve 0.8 discovery power; IVA.**
Rare, medium-effect-size alleles (scenario D). The diagnosed-individuals-versus-same-age-unaffected-population curve continues to rise steeply in the IVA scenario. A sample of 9 out of 25 SNPs; MAF = minor (risk) allele frequency; OR = risk odds ratio.

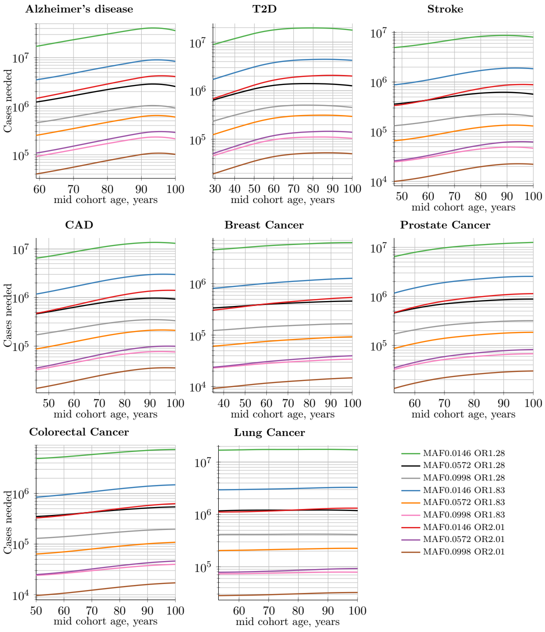


**Supplemental Fig. 11. Number of cases needed to achieve 0.8 discovery power; cohort simulation.**
Rare medium-effect-size alleles (scenario D). The cohort curve due to the accumulative cases diagnosed at younger ages with an averaged control polygenic risk score and mortality begins at the same necessary-cases number as IVA but rises more slowly and levels out at older ages. A sample of 9 out of 25 SNPs; MAF = minor (risk) allele frequency; OR = risk odds ratio.

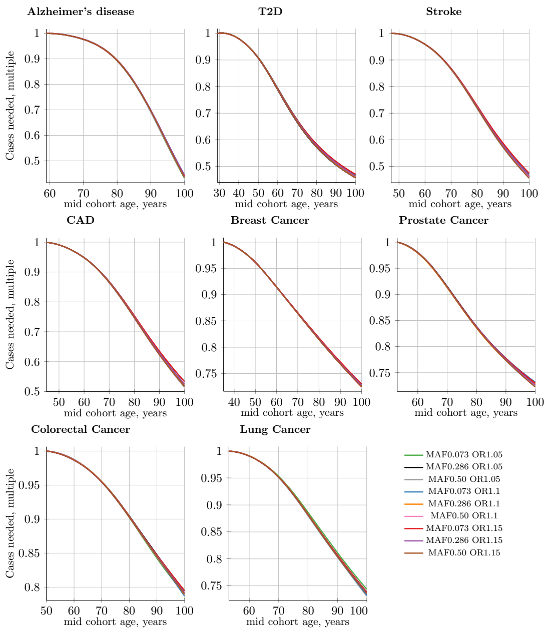


**Supplemental Fig. 12. Multiple of the decline in the number of cases needed for 0.8 discovery power in a cohort study using progressively older control cohorts compared to a fixed-age young-cases cohort.**
Cases' mid-cohort age is leftmost age (youngest plot point); control mid-cohort ages are incremental ages. The number of cases needed for 0.8 discovery power is smaller when older controls are used, particularly for LODs with the highest heritability and incidence. Common, low-effect-size alleles (scenario A). A sample of 9 out of 25 SNPs; MAF = minor (risk) allele frequency; OR = risk odds ratio.

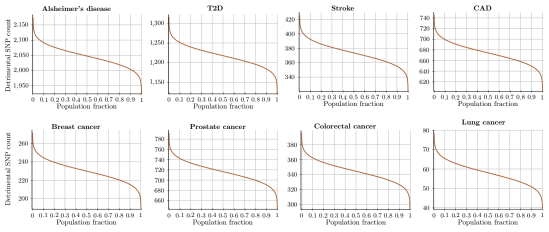


**Supplemental Fig. 13. Population distribution of malignant variants for common, low-effect-size genetic architecture.**
Based on initial heritability, the individuals in a population carry a relatively high number of malignant, low-effect alleles, resulting in the combined LOD PRS.

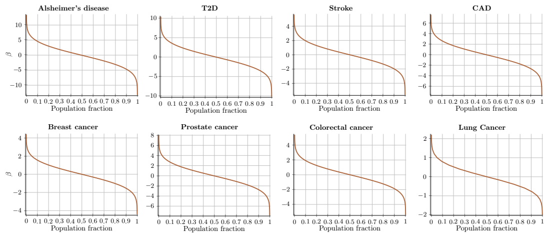


**Supplemental Fig. 14. Population distribution of PRSs for common, low-effect-size genetic architecture.**
$\beta = log(OddsRatio)$ normalized to population mean.

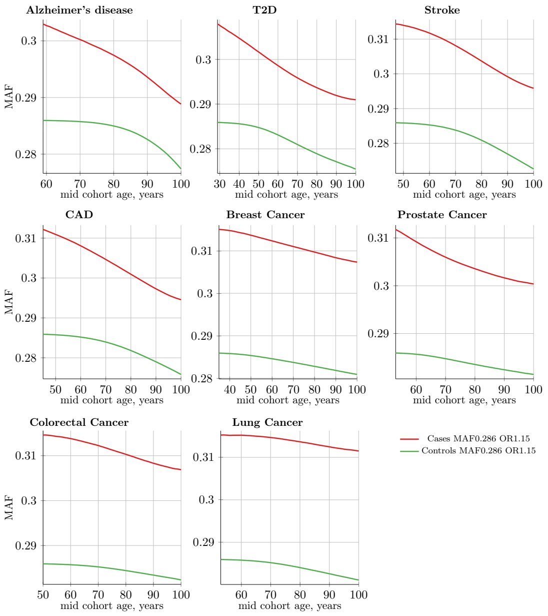


**Supplemental Fig. 15. Absolute magnitude change in MAF (minor allele frequency) with age for cases and controls; cohort simulation.**

Common, low-effect-size alleles (scenario A), all plots show MAF = 0.286 and OR = 1.15 allele. Change in the absolute magnitude of each allele frequency value is relatively small with age progression. GWAS discovery power is a function of the difference in allele frequency between cases and controls. Rarer and lower-effect-size (OR) alleles are characterized by a lower change in absolute and relative MAF with cohort age progression.

## References


A. Ahmad, N. Ormiston-Smith, and P. Sasieni. Trends in the lifetime risk of developing cancer in great britain: comparison of risk for those born from 1930 to 1960. *British journal of cancer*, 112(5):943, 2015.

S. A. Allport, N. Kikah, N. A. Saif, F. Ekokobe, and F. D. Atem. Parental age of onset of cardiovascular disease as a predictor for offspring age of onset of cardiovascular disease. *PloS one*, 11(12):e0163334, 2016.

P. Almgren, M. Lehtovirta, B. Isomaa, L. Sarelin, M. Taskinen, V. Lyssenko, T. Tuomi, L. Groop, and B. S. Group. Heritability and familiality of type 2 diabetes and related quantitative traits in the botnia study. *Diabetologia*, 54(11):2811, 2011.

A. O. Amoako and G. G. A. Pujalte. Osteoarthritis in young, active, and athletic individuals. *Clinical Medicine Insights: Arthritis and Musculoskeletal Disorders*, 7:CMAMD–S14386, 2014.

A. Antoniou, P. D. Pharoah, S. Narod, H. A. Risch, J. E. Eyfjord, J. L. Hopper, N. Loman, H. Olsson, O. Johannsson, Å. Borg, et al. Average risks of breast and ovarian cancer associated with brca1 or brca2 mutations detected in case series unselected for family history: a combined analysis of 22 studies. *The American Journal of Human Genetics*, 72(5):1117–1130, 2003.

H. J. Aparicio and S. Seshadri. Familial occurrence and heritability of stroke. In *Stroke Genetics*, pages 9–20. Springer, 2017.

S. Bevan, M. Traylor, P. Adib-Samii, R. Malik, N. L. Paul, C. Jackson, M. Farrall, P. M. Rothwell, C. Sudlow, M. Dichgans, et al. Genetic heritability of ischemic stroke and the contribution of previously reported candidate gene and genomewide associations. *Stroke*, 43(12):3161–3167, 2012.

N. Binder and M. Schumacher. Incidence of dementia over three decades in the framingham heart study. *The New England journal of medicine*, 375(1):92–93, 2016.

M. W. Boehme, G. Buechele, J. Frankenhauser-Mannuss, J. Mueller, D. Lump, B. O. Boehm, and D. Rothenbacher. Prevalence, incidence and concomitant co-morbidities of type 2 diabetes mellitus in south western germany-a retrospective cohort and case control study in claims data of a large statutory health insurance. *BMC Public Health*, 15(1):855, 2015.

R. Brookmeyer, S. Gray, and C. Kawas. Projections of alzheimer's disease in the united states and the public health impact of delaying disease onset. *American journal of public health*, 88 (9):1337–1342, 1998.

Cancer Statistics for the UK. *Available at http://www.cancerresearchuk.org/health-professional/cancer-statistics-for-the-uk (accessed November 10, 2018)*, 2018.

S. R. Carr, W. Akerley, M. Hashibe, and L. A. Cannon-Albright. Evidence for a genetical contribution to non-smoking-related lung cancer. *Thorax*, pages thoraxjnl–2014, 2015.

Y. Davidson, L. Gibbons, A. Pritchard, J. Hardicre, J. Wren, C. Stopford, C. Julien, J. Thompson, A. Payton, S. M. Pickering-Brown, et al. Apolipoprotein e $\varepsilon 4$ allele frequency and age at onset of alzheimer's disease. *Dementia and geriatric cognitive disorders*, 23(1):60–66, 2007.

J. M. de Miguel-Yanes, P. Shrader, M. J. Pencina, C. S. Fox, A. K. Manning, R. W. Grant, J. Dupuis, J. C. Florez, R. B. D'agostino, L. A. Cupples, et al. Genetic risk reclassification for type 2 diabetes by age below or above 50 years using 40 type 2 diabetes risk single nucleotide polymorphisms. *Diabetes care*, 34 (1):121–125, 2011.

R. M. de Voer, M.-M. Hahn, R. D. Weren, A. R. Mensenkamp, C. Gilissen, W. A. van Zelst-Stams, L. Spruijt, C. M. Kets, J. Zhang, and H. Venselaar. Identification of novel candidate genes for early-onset colorectal cancer susceptibility. *PLoS genetics*, 12(2):e1005880, 2016.

W. J. Devan, G. J. Falcone, C. D. Anderson, J. M. Jagiella, H. Schmidt, B. M. Hansen, J. Jimenez-Conde, E. Giralt-Steinhauer, E. Cuadrado-Godia, C. Soriano, et al. Heritability estimates identify a substantial genetic contribution to risk and outcome of intracerebral hemorrhage. *Stroke*, 44(6): 1578–1583, 2013.

Diapedia: Epidemiology of type 2 diabetes. *Available at https://www.diapedia.org/type-2-diabetes-mellitus/3104287123/epidemiology-of-type-2-diabetes (accessed November 10, 2018)*, 2016.

R. Eeles, A. A. Al Olama, S. Berndt, F. Wiklund, D. V. Conti, M. Ahmed, S. Benlloch, D. Easton, P. Kraft, S. J. Chanock, et al. Prostate cancer meta-analysis from more than 145,000 men to identify 65 novel prostate cancer susceptibility loci. *Journal of Clinical Oncology*, 2017.

C. E. Elks, M. Den Hoed, J. H. Zhao, S. J. Sharp, N. J. Wareham, R. J. Loos, and K. K. Ong. Variability in the heritability of body mass index: a systematic review and meta-regression. *Frontiers in endocrinology*, 3:29, 2012.

D. Falconer. The inheritance of liability to diseases with variable age of onset, with particular reference to diabetes mellitus. *Annals of human genetics*, 31(1):1–20, 1967.

L. A. Farrer, L. A. Cupples, J. L. Haines, B. Hyman, W. A. Kukull, R. Mayeux, R. H. Myers, M. A. Pericak-Vance, N. Risch, and C. M. Van Duijn. Effects of age, sex, and ethnicity on the association between apolipoprotein e genotype and alzheimer disease: a meta-analysis. *Jama*, 278(16):1349–1356, 1997.

D. Ford, D. Easton, M. Stratton, S. Narod, D. Goldgar, P. Devilee, D. Bishop, B. Weber, G. Lenoir, J. Chang-Claude, et al. Genetic heterogeneity and penetrance analysis of the brca1 and brca2 genes in breast cancer families. *The American Journal of Human Genetics*, 62(3):676–689, 1998.

C. Fuchsberger, J. Flannick, T. M. Teslovich, A. Mahajan, V. Agarwala, K. J. Gaulton, . C. Ma, P. Fontanillas, L. Moutsianas, D. J. McCarthy, et al. The genetic architecture of type 2 diabetes. *Nature*, 536(7614):41–47, 2016.

M. Gatz, C. A. Reynolds, L. Fratiglioni, B. Johansson, J. A. Mortimer, S. Berg, A. Fiske, and N. L. Pedersen. Role of genes and environments for explaining alzheimer disease. *Archives of general psychiatry*, 63(2):168–174, 2006.

R. E. Graff, S. Möller, M. N. Passarelli, J. S. Witte, A. Skytthe, K. Christensen, Q. Tan, H.-O. Adami, K. Czene, and J. R. Harris. Familial risk and heritability of colorectal cancer in the nordic twin study of cancer. *Clinical Gastroenterology and Hepatology*, 15(8):1256–1264, 2017.

H. Grönberg. Prostate cancer epidemiology. *The Lancet*, 361 (9360):859–864, 2003.

A. Guedj, A. Geiger-Maor, E. Galun, H. Amsalem, and J. Rachmilewitz. Early age decline in dna repair capacity in the liver: in depth profile of differential gene expression. *Aging (Albany NY)*, 8(11):3131, 2016.

B. Haley. Hereditary breast cancer: the basics of brca and beyond. *UT Southwestern Medical Center. Internal Medicine Grand Rounds*, 2016.

J. Hjelmborg, T. Korhonen, K. Holst, A. Skytthe, E. Pukkala, J. Kutschke, J. R. Harris, L. A. Mucci, K. Christensen, K. Czene, et al. Lung cancer, genetic predisposition and smoking: the nordic twin study of cancer. *Thorax*, pages thoraxjnl–2015, 2016.

J. B. Hjelmborg, T. Scheike, K. Holst, A. Skytthe, K. L. Penney, R. E. Graff, E. Pukkala, K. Christensen, H.-O. Adami, N. V. Holm, et al. The heritability of prostate cancer in the nordic twin study of cancer. *Cancer Epidemiology and Prevention Biomarkers*, 23(11):2303–2310, 2014.

S. H. Jee, I. Suh, S. Y. Won, and M. Y. Kim. Familial correlation and heritability for cardiovascular risk factors. *Yonsei medical journal*, 43(2):160–164, 2002.

S. Jiao, U. Peters, S. Berndt, H. Brenner, K. Butterbach, B. J. Caan, C. S. Carlson, A. T. Chan, J. Chang-Claude, S. Chanock, et al. Estimating the heritability of colorectal cancer. *Human molecular genetics*, 23(14):3898–3905, 2014.

M. Kanwal, X.-J. Ding, and Y. Cao. Familial risk for lung cancer. *Oncology Letters*, 13(2):535–542, 2017.

D. Krewski, J. H. Lubin, J. M. Zielinski, M. Alavanja, V. S. Catalan, R. W. Field, J. B. Klotz, E. G. Létourneau, C. F. Lynch, J. I. Lyon, et al. Residential radon and risk of lung cancer: a combined analysis of 7 north american case-control studies. *Epidemiology*, 16(2):137–145, 2005.

K. B. Kuchenbaecker, J. L. Hopper, D. R. Barnes, K.-A. Phillips, T. M. Mooij, M.-J. Roos-Blom, S. Jervis, F. E. Van Leeuwen, R. L. Milne, and N. Andrieu. Risks of breast, ovarian, and contralateral breast cancer for brca1 and brca2 mutation carriers. *Jama*, 317(23):2402–2416, 2017.

J. Lecarpentier, V. Silvestri, K. B. Kuchenbaecker, D. Barrowdale, J. Dennis, L. McGuffog, P. Soucy, G. Leslie, P. Rizzolo, and A. S. Navazio. Prediction of breast and prostate cancer risks in male brca1 and brca2 mutation carriers using polygenic risk scores. *Journal of Clinical Oncology*, 35(20):2240–2250, 2017.

Lifetime Risk of Developing or Dying From Cancer. *American Cancer Society. Available at https://www.cancer.org/cancer/cancer-basics/lifetime-probability-of-developing-or-dying-from-cancer.html (accessed November 10, 2018)*, 2018.

D. M. Lloyd-Jones, E. P. Leip, M. G. Larson, R. B. d'Agostino, A. Beiser, P. W. Wilson, P. A. Wolf, and D. Levy. Prediction of lifetime risk for cardiovascular disease by risk factor burden at 50 years of age. *Circulation*, 113(6):791–798, 2006.

J. Loughlin. Genetic contribution to osteoarthritis development: current state of evidence. *Current opinion in rheumatology*, 27 (3):284, 2015.

P. C. Lyra-Junior, N. G. Tessarollo, I. S. Guimarães, T. B. Henriques, D. Z. dos Santos, M. L. de Souza, V. H. M. Marques, L. F. de Oliveira, K. V. Siqueira, I. V. Silva, et al. Gwas in breast cancer. In *Breast Cancer-From Biology to Medicine*, pages 99–117. InTech, 2017.

S. S. Mahmood, D. Levy, R. S. Vasan, and T. J. Wang. The framingham heart study and the epidemiology of cardiovascular disease: a historical perspective. *The Lancet*, 383(9921):999–1008, 2014.

J. Malhotra, M. Malvezzi, E. Negri, C. La Vecchia, and P. Boffetta. Risk factors for lung cancer worldwide. *European Respiratory Journal*, pages ERJ–00359, 2016.

N. Mancuso, N. Rohland, K. A. Rand, A. Tandon, A. Allen, D. Quinque, S. Mallick, H. Li, A. Stram, and X. Sheng. The contribution of rare variation to prostate cancer heritability. *Nature genetics*, 48(1):30, 2016.

A. Marusyk and J. DeGregori. Declining cellular fitness with age promotes cancer initiation by selecting for adaptive oncogenic mutations. *Biochimica et Biophysica Acta (BBA)-Reviews on Cancer*, 1785(1):1–11, 2008.

N. Mavaddat, A. C. Antoniou, D. F. Easton, and M. Garcia-Closas. Genetic susceptibility to breast cancer. *Molecular oncology*, 4 (3):174–191, 2010.

K. Michailidou, S. Lindström, J. Dennis, J. Beesley, S. Hui, S. Kar, A. Lemaçon, P. Soucy, D. Glubb, A. Rostamianfar, et al. Association analysis identifies 65 new breast cancer risk loci. *Nature*, 551(7678):92, 2017.

S. Möller, L. A. Mucci, J. R. Harris, T. Scheike, K. Holst, U. Halekoh, H.-O. Adami, K. Czene, K. Christensen, N. V. Holm, et al. The heritability of breast cancer among women in the nordic twin study of cancer. *Cancer Epidemiology and Prevention Biomarkers*, 25(1):145–150, 2016.

L. A. Mucci, J. B. Hjelmborg, J. R. Harris, K. Czene, D. J. Havelick, T. Scheike, R. E. Graff, K. Holst, S. Möller, R. H. Unger, et al. Familial risk and heritability of cancer among twins in nordic countries. *Jama*, 315(1):68–76, 2016.

S. L. Murphy, J. Xu, K. D. Kochanek, S. C. Curtin, and E. Arias. Mortality in the united states, 2016. *NCHS Data Brief, no 293*, 293, 2017.

A. C. Naj and G. D. Schellenberg. Genomic variants, genes, and pathways of alzheimer's disease: An overview. *American Journal of Medical Genetics Part B: Neuropsychiatric Genetics*, 174 (1):5–26, 2017.

P. Nelson and J. Masel. Intercellular competition and the inevitability of multicellular aging. *Proceedings of the National Academy of Sciences*, page 201618854, 2017.

M. Nielsen, C. Andersson, T. A. Gerds, P. K. Andersen, T. B. Jensen, L. Køber, G. Gislason, and C. Torp-Pedersen. Familial clustering of myocardial infarction in first-degree relatives: a nationwide study. *European heart journal*, 34(16):1198–1203, 2013.

T. J. Polderman, B. Benyamin, C. A. De Leeuw, P. F. Sullivan, A. Van Bochoven, P. M. Visscher, and D. Posthuma. Meta-analysis of the heritability of human traits based on fifty years of twin studies. *Nature genetics*, 47(7):702, 2015.

P. Poulsen, K. O. Kyvik, A. Vaag, and H. Beck-Nielsen. Heritability of type ii (non-insulin-dependent) diabetes mellitus and abnormal glucose tolerance–a population-based twin study. *Diabetologia*, 42(2):139–145, 1999.

S. H. Ralston and A. G. Uitterlinden. Genetics of osteoporosis. *Endocrine reviews*, 31(5):629–662, 2010.

F. Ribezzo, Y. Shiloh, and B. Schumacher. Systemic dna damage responses in aging and diseases. In *Seminars in cancer biology*, volume 37, pages 26–35. Elsevier, 2016.

H. A. Risch, J. R. McLaughlin, D. E. Cole, B. Rosen, L. Bradley, I. Fan, J. Tang, S. Li, S. Zhang, and P. A. Shaw. Population brca1 and brca2 mutation frequencies and cancer penetrances: a kin–cohort study in ontario, canada. *Journal of the National Cancer Institute*, 98(23):1694–1706, 2006.

P. Rothwell, A. Coull, L. Silver, J. Fairhead, M. Giles, C. Lovelock, J. Redgrave, L. Bull, S. Welch, and F. Cuthbertson. Population-based study of event-rate, incidence, case fatality, and mortality for all acute vascular events in all arterial territories (oxford vascular study). *The Lancet*, 366(9499):1773–1783, 2005.

P. Sasieni, J. Shelton, N. Ormiston-Smith, C. Thomson, and P. Silcocks. What is the lifetime risk of developing cancer?: the effect of adjusting for multiple primaries. *British journal of cancer*, 105(3):460, 2011.

S. L. Schmit, F. R. Schumacher, C. K. Edlund, D. V. Conti, U. Ihenacho, P. Wan, D. Van Den Berg, G. Casey, B. K. Fortini, H.-J. Lenz, et al. Genome-wide association study of colorectal cancer in hispanics. *Carcinogenesis*, 37(6):547–556, 2016.

U. Schulz, E. Flossmann, and P. Rothwell. Heritability of ischemic stroke in relation to age, vascular risk factors, and subtypes of incident stroke in population-based studies. *Stroke*, 35(4): 819–824, 2004.

S. Seshadri, A. Beiser, A. Pikula, J. J. Himali, M. Kelly-Hayes, S. Debette, A. L. DeStefano, J. R. Romero, C. S. Kase, and P. A. Wolf. Parental occurrence of stroke and risk of stroke in their children: the framingham study. *Circulation*, 121(11): 1304–1312, 2010.

J. R. Shaffer, C. M. Kammerer, J. M. Bruder, S. A. Cole, T. D. Dyer, L. Almasy, J. W. MacCluer, J. Blangero, R. L. Bauer, and B. D. Mitchell. Genetic influences on bone loss in the san antonio family osteoporosis study. *Osteoporosis international*, 19(12): 1759–1767, 2008.

L. Shen and J. Jia. An overview of genome-wide association studies in alzheimer's disease. *Neuroscience bulletin*, 32(2): 183–190, 2016.

L. Shi, M. Zheng, J. Hou, B. Zhu, and X. Wang. Regulatory roles of epigenetic modulators, modifiers and mediators in lung cancer. In *Seminars in cancer biology*, volume 42, pages 4–12. Elsevier, 2017.

S. G. Skousgaard, J. Hjelmborg, A. Skytthe, L. P. A. Brandt, S. Möller, and S. Overgaard. Probability and heritability estimates on primary osteoarthritis of the hip leading to total hip arthroplasty: a nationwide population based follow-up study in danish twins. *Arthritis research & therapy*, 17(1):336, 2015.

S. G. Skousgaard, A. Skytthe, S. Möller, S. Overgaard, and L. Brandt. Sex differences in risk and heritability estimates on primary knee osteoarthritis leading to total knee arthroplasty: a nationwide population based follow up study in danish twins. *Arthritis research & therapy*, 18:46–46, 2016.

T. D. Spector and A. J. MacGregor. Risk factors for osteoarthritis: genetics. *Osteoarthritis and cartilage*, 12:39–44, 2004.

P. J. Talmud, J. A. Cooper, R. W. Morris, F. Dudbridge, T. Shah, J. Engmann, C. Dale, J. White, S. McLachlan, D. Zabaneh, et al. Sixty-five common genetic variants and prediction of type 2 diabetes. *Diabetes*, page DB_141504, 2014.

L. Tan, J.-T. Yu, W. Zhang, Z.-C. Wu, Q. Zhang, Q.-Y. Liu, W. Wang, H.-F. Wang, X.-Y. Ma, and W.-Z. Cui. Association of gwas-linked loci with late-onset alzheimer's disease in a northern han chinese population. *Alzheimer's & dementia*, 9(5):546–553, 2013.

C. Tomasetti and B. Vogelstein. Variation in cancer risk among tissues can be explained by the number of stem cell divisions. *Science*, 347(6217):78–81, 2015.

P. C. Walsh. The search for the missing heritability of prostate cancer. *European urology*, 72(5):657–659, 2017.

D. C. Wang and X. Wang. Tomorrow's genome medicine in lung cancer. In *Seminars in cancer biology*, volume 42, pages 39–43. Elsevier, 2017.

S. C. Warner and A. M. Valdes. The genetics of osteoarthritis: A review. *Journal of Functional Morphology and Kinesiology*, 1 (1):140–153, 2016.

J. L. Weissfeld, Y. Lin, H.-M. Lin, B. F. Kurland, D. O. Wilson, C. R. Fuhrman, A. Pennathur, M. Romkes, T. Nukui, and J.-M. Yuan. Lung cancer risk prediction using common snps located in gwas-identified susceptibility regions. *Journal of Thoracic Oncology*, 10(11):1538–1545, 2015.

A. Wienke, N. V. Holm, A. Skytthe, and A. I. Yashin. The heritability of mortality due to heart diseases: a correlated frailty model applied to danish twins. *Twin Research and Human Genetics*, 4(4):266–274, 2001.

X. Wu and J. Gu. Heritability of prostate cancer: a tale of rare variants and common single nucleotide polymorphisms. *Annals of translational medicine*, 4(10), 2016.

Y.-T. Wu, A. S. Beiser, M. M. Breteler, L. Fratiglioni, C. Helmer, H. C. Hendrie, H. Honda, M. A. Ikram, K. M. Langa, and A. Lobo. The changing prevalence and incidence of dementia over time - current evidence. *Nature Reviews Neurology*, 2017.

I. A. Yang, J. W. Holloway, and K. M. Fong. Genetic susceptibility to lung cancer and co-morbidities. *Journal of thoracic disease*, 5(Suppl 5):S454, 2013.

S. Zdravkovic, A. Wienke, N. Pedersen, M. Marenberg, A. Yashin, and U. De Faire. Heritability of death from coronary heart disease: a 36-year follow-up of 20 966 swedish twins. *Journal of internal medicine*, 252(3):247–254, 2002.
